# A red nucleus–VTA glutamate pathway underlies exercise reward and the therapeutic effect of exercise on cocaine use

**DOI:** 10.1126/sciadv.abo1440

**Published:** 2022-09-02

**Authors:** Yi He, Graziella Madeo, Ying Liang, Cindy Zhang, Briana Hempel, Xiaojie Liu, Lianwei Mu, Shui Liu, Guo-Hua Bi, Ewa Galaj, Hai-Ying Zhang, Hui Shen, Ross A. McDevitt, Eliot L. Gardner, Qing-song Liu, Zheng-Xiong Xi

**Affiliations:** ^1^ Molecular Targets and Medications Discovery Branch, National Institute on Drug Abuse, Intramural Research Program, Baltimore, MD 21224, USA.; ^2^ Cellular Neurobiology Branch, National Institute on Drug Abuse, Intramural Research Program, Baltimore, MD 21224, USA.; ^3^ Department of Pharmacology and Toxicology, Medical College of Wisconsin, Milwaukee, WI 53226, USA.; ^4^ Section on Molecular Neuroscience, National Institute of Mental Health, Bethesda, MD 20892, USA.; ^5^ Comparative Medicine Section, National Institute on Aging, Intramural Research Program, Baltimore, MD 21224, USA.

## Abstract

Physical exercise is rewarding and protective against drug abuse and addiction. However, the neural mechanisms underlying these actions remain unclear. Here, we report that long-term wheel-running produced a more robust increase in c-fos expression in the red nucleus (RN) than in other brain regions. Anatomic and functional assays demonstrated that most RN magnocellular portion (RNm) neurons are glutamatergic. Wheel-running activates a subset of RNm glutamate neurons that project to ventral tegmental area (VTA) dopamine neurons. Optogenetic stimulation of this pathway was rewarding, as assessed by intracranial self-stimulation and conditioned place preference, whereas optical inhibition blocked wheel-running behavior. Running wheel access decreased cocaine self-administration and cocaine seeking during extinction. Last, optogenetic stimulation of the RNm-to-VTA glutamate pathway inhibited responding to cocaine. Together, these findings indicate that physical exercise activates a specific RNm-to-VTA glutamatergic pathway, producing exercise reward and reducing cocaine intake.

## Teaser

Physical exercise activates a glutamate pathway that links motor and reward circuits, producing reward and anticocaine effects.

## INTRODUCTION

Physical exercise is rewarding and antidepressive (*1*). Long-distance runners often describe the “runner’s high” as a pleasant feeling of euphoria and relaxation similar to that produced by drugs of abuse (*2*, *3*). Physical exercise is also protective against the development of substance use disorders and reduces the risk of drug abuse (*4*). In preclinical studies, rats and mice choose to run spontaneously on running wheels in the absence of any explicit reward (e.g., food) and rapidly learn to lever-press for access to a running wheel (*3*, *5*). They also develop conditioned place preference (CPP) for environments associated with wheel-running (*6*, *7*). In addition, wheel-running is reported to reduce drug self-administration in laboratory animals (*4*, *8*). However, the neural mechanisms underlying exercise reward and its therapeutic effects against drug abuse are poorly understood.

Mesolimbic dopamine (DA) activity mediates the reinforcing properties of natural rewards (e.g., palatable food and sex) and drugs of abuse (*9*). Therefore, it has been proposed that exercise may activate the mesolimbic DA system, producing rewarding effects (*10*). Voluntary wheel-running has been reported to increase striatal DA levels (*11*) while decreasing sensitivity to drugs of abuse in rats (*12*, *13*). This action could be mediated indirectly by activation of the endogenous opioid system (*14*, *15*), given that exercise has been shown to increase blood levels of β-endorphin in mice and humans (*15*, *16*). Prior exposure to wheel-running produces cross-tolerance to the rewarding effects of morphine (*17*), while blockade of opioid receptors by naloxone attenuates the reinforcing effects of exercise in experimental animals (*18*, *19*), suggesting that endogenous opioids may mediate physical exercise’s reinforcing effect. However, little is known as to how physical exercise increases endogenous opioid release. In addition, it has been reported that intense physical exercise increases blood endocannabinoid [anandamide (AEA)] levels (*20*, *21*), suggesting possible mechanistic involvement of endocannabinoids in exercise reward (*22*, *23*). This is supported by the finding that genetic deletion of the cannabinoid CB1 receptor reduces voluntary wheel-running (*24*) but not supported by the finding that high runner (HR) mice display lower circulating levels of 2-arachidonoylglycerol (2-AG) and AEA than control mice (*25*) and the finding that both cannabinoid receptor agonists (WIN55,212-2, 2-AG) and antagonists (rimonabant) reduce wheel-running behavior in selectively bred HR mice and control mice (*26*, *27*). It is also unknown how physical exercise increases endocannabinoid release.

Here, we took an approach to identify and characterize the specific neural circuitry that may underlie exercise reward. We hypothesized that physical exercise might initially stimulate a motor-related neural circuit, which subsequently activates the mesolimbic DA reward system. In other words, long-term exercise may act on an unidentified neural circuit that integrates the motor-regulation system and the brain reward system. Identification of such circuitry would appear crucial for understanding why physical exercise is rewarding and how it produces therapeutic benefits against drug abuse and addiction. Using multidisciplinary approaches, we identified a glutamatergic pathway from the red nucleus (RN) to the ventral tegmental area (VTA) that at least in part underlies exercise reward.

## RESULTS

### Wheel-running exercise activates neurons in the RN and VTA

**Fig. 1.**
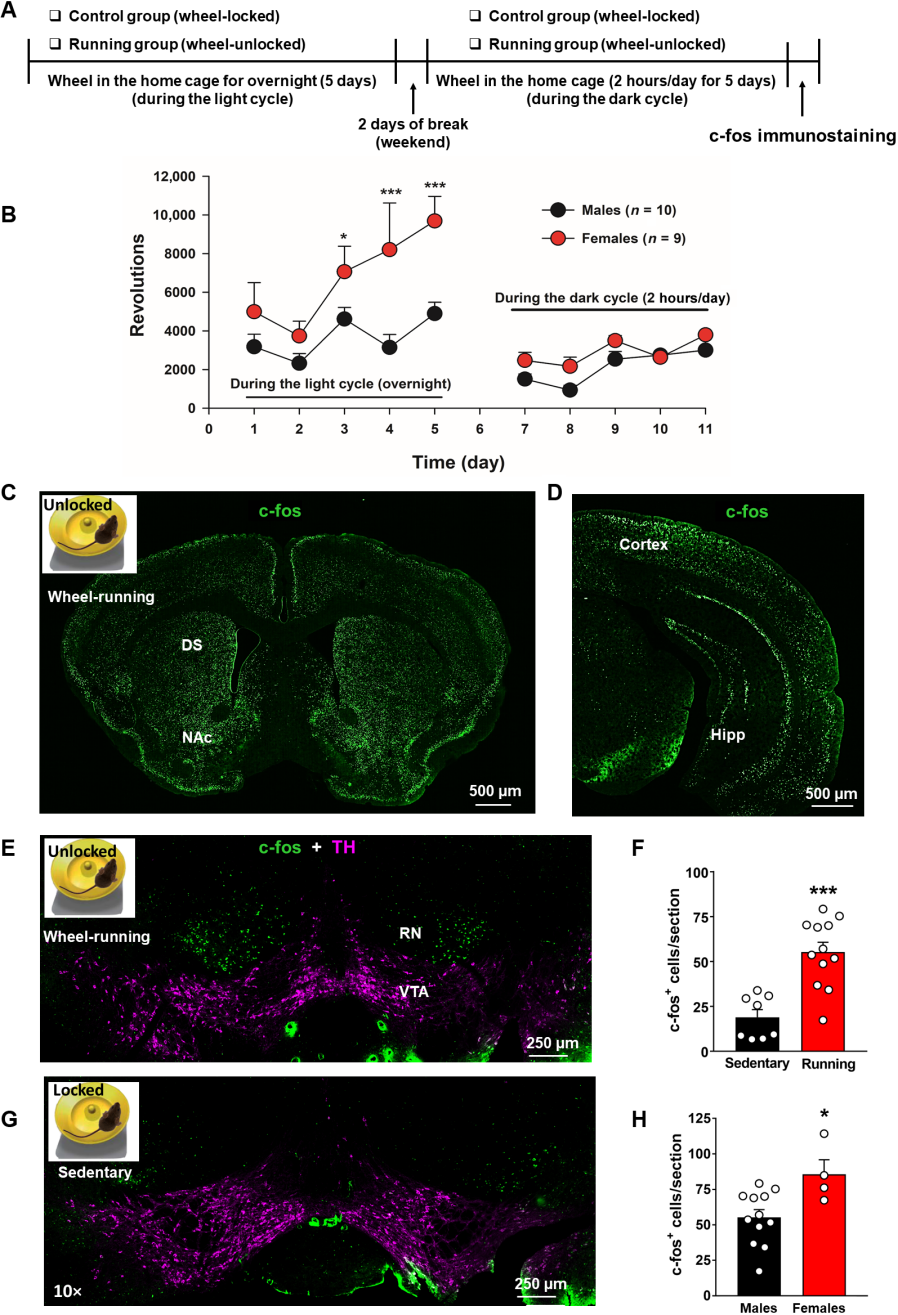
Effects of wheel-running on c-fos expression in the mouse brain. (**A**) Procedural timeline. (**B**) Wheel-running performance during the initial 5 days of overnight habituation training (~12 hours per night) during the light cycle and 5 days of daily 2-hour running during the dark cycle. There was no running in the sedentary control mice since the wheels were locked. (**C** to **E**) Representative images showing wheel-running–induced c-fos expression in the cortex and striatum (C), hippocampus (Hipp) (D), and RN (E). (**F**) Average cell counting across animals indicating that wheel-running produced a robust increase in c-fos expression in the RN of male mice compared to the sedentary control group (***P < 0.001). (**G**) Representative image indicating that the c-fos level is very low in a sedentary control mouse. (**H**) Average cell counting of c-fos expression in males and females after wheel-running exercise (*P < 0.05). Scale bars, 500 μm (C and D) and 250 μm (E and G) (also see figs. S1 and S2).

Since c-fos is transiently expressed in response to neuronal stimulation (*28*), we first examined c-fos expression following voluntary wheel-running to determine which brain regions are activated by physical exercise. Given that prolonged wheel-running is required to observe the rewarding and antidepressive effects of exercise in mice (*1*, *3*), we used a 10-day wheel-running procedure to observe exercise-induced c-fos expression in the brain (Fig. 1A). During the first 5 days, the running group of mice was allowed access to unlocked running wheels in their home cages for free running during the light cycle (12 hours) in a test room with a reverse light-dark cycle. After a 2-day break (weekend), the animals were provided running wheels in their home cage for 2 hours per day across five consecutive days during the dark cycle in the same test room (Fig. 1A). Immediately after the final 2-hour running session, mice were euthanized and decapitated for c-fos examination. The sedentary control group of mice followed the same procedures, except the wheels were locked (Fig. 1A). Figure 1B shows running performance and demonstrates that females ran more than males during the first 5 days of overnight running. However, there was no difference between males and females during the last 5 days of daily 2-hour running during the dark cycle. A two-way analysis of variance (ANOVA) for repeated measures (RM) over time revealed a significant sex main effect (*F*_1,17_ = 9.13, *P* < 0.01), time main effect (*F*_9,153_ = 12.68, *P* < 0.001), and sex × time interaction (*F*_9,153_ = 2.71, *P* < 0.05). Post hoc tests for multiple group comparisons revealed significant sex differences on days 3 to 5 during the overnight training sessions, but no sex differences during the second phase of daily 2-hour training sessions (Fig. 1B). Figure 1 (C to E and G) shows representative c-fos immunostaining in both wheel-running and sedentary control groups of mice. Wheel-running increased c-fos expression in multiple brain regions, including the motor cortex, hippocampus, and dorsal striatum (Fig. 1, C to E), as compared to the sedentary control mice (Fig. 1G and fig. S1). However, the most robust increase in c-fos response occurred in the RN, particularly in the magnocellular portion of the RN (RNm) (Fig. 1, E and G). Figure S1 shows c-fos expression in a series of slices from the rostral to caudal RN, illustrating robust increases in c-fos expression in the RNm in a wheel-running mouse compared to a control mouse. A moderate increase in c-fos expression was also observed in a subset (~10%) of VTA DA neurons (fig. S2). An unbiased cell counting analysis indicated that prolonged daily wheel-running significantly increased c-fos expression in RNm neurons compared to sedentary control mice (unpaired *t* test, *P* < 0.001; Fig. 1F). In addition, female mice displayed a significantly higher c-fos response to wheel-running than males (unpaired *t* test, *P* < 0.05; Fig. 1H). This is consistent with our behavioral finding that females run more than males (Fig. 1B).

### Most neurons in the RNm are glutamatergic

The RN consists of the dorsolateral parvocellular part (RNp) and the RNm (*29*). The giant size of magnocellular neurons is a landmark feature of the RNm. To determine the phenotypes of RN neurons that are activated by wheel-running, we examined cellular distributions of γ-aminobutyric acid (GABA) neurons versus glutamate neurons in both the RNp and RNm. Figure S3 shows GABAergic and glutamatergic neuron distributions in a series of slices from the rostral to the caudal RN. In this experiment, the AAV that carries a gene sequence to express an enhanced yellow fluorescent protein (AAV-DIO-eYFP) was microinjected into the middle RN at bregma level −3.40 mm to selectively express eYFP in GABA neurons in Vgat-IRES-Cre (Vgat-Cre) mice (fig. S3A). We found that most neurons in the RNp are GABAergic, with densities progressively decreasing toward the caudal RNm (fig. S3A). Notably, GABAergic neurons are almost undetectable in the RNm.

**Fig. 2.**
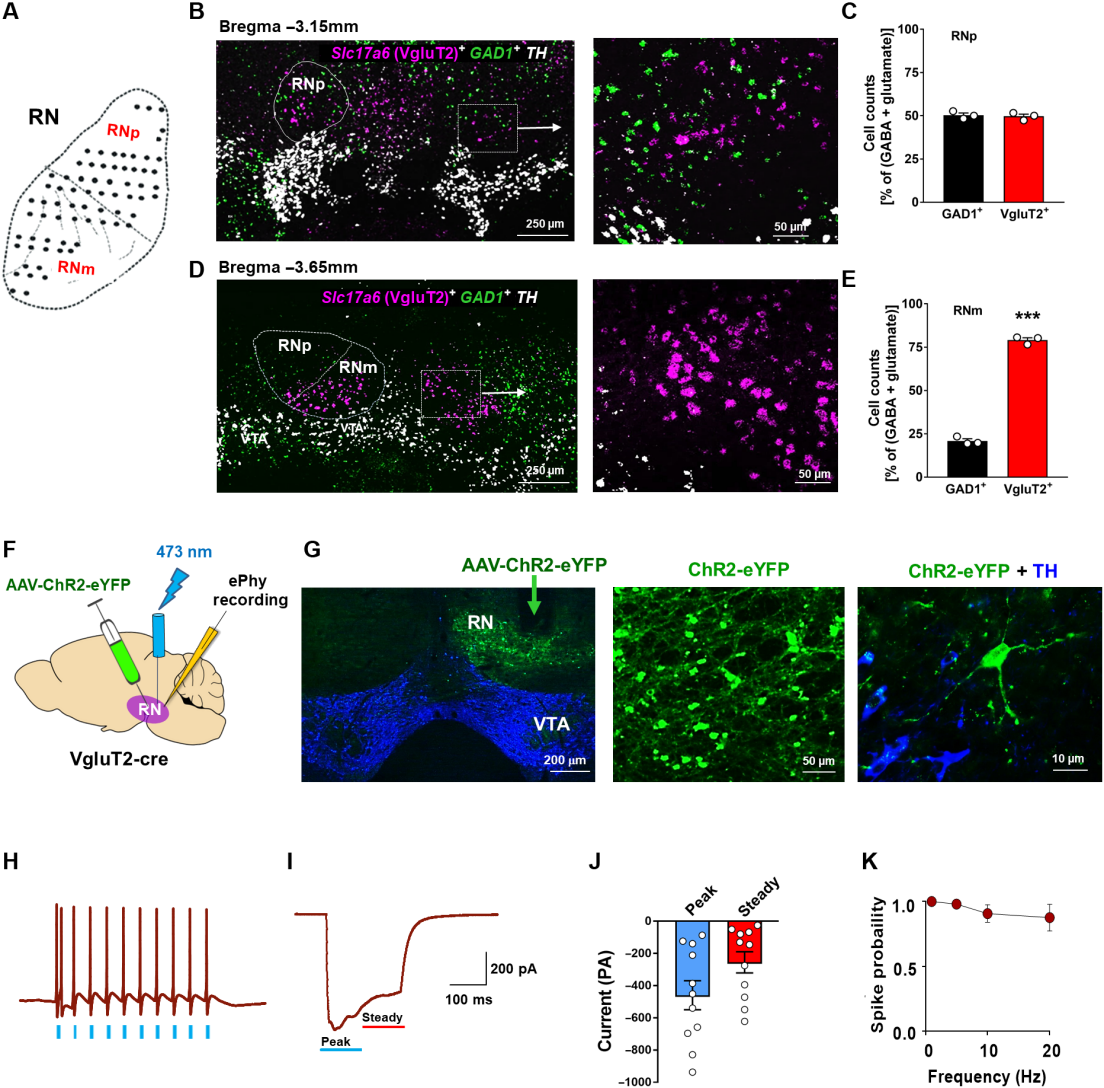
Identification of phenotypes of neurons in the RN. (**A**) A diagram of the red nucleus (RN), illustrating that the RN consists of a rostral paracellular part (RNp) and a caudal magnocellular part (RNm) in mice. (**B**) Representative RNAscope ISH images showing *Slc17a6* (VgluT2)^+^ glutamate neurons (magenta) and *GAD1*^+^ GABA neurons (green) in the RNp and TH^+^ DA neurons (white) in the VTA. (**C**) Quantitative cell counting data indicating that the RNp is composed of GABAergic neurons (~50%) and glutamatergic neurons (~50%). (**D**) Representative RNAscope ISH images showing VgluT2^+^ glutamate neurons and GAD1^+^ GABA neurons in the RNm and TH^+^ DA neurons in the VTA. (**E**) Quantitative cell counting data indicating that most RNm neurons are VgluT2^+^ glutamatergic (>75%). (**F**) Schematic diagram of the electrophysiological experiments and representative images, showing that AAV-ChR2-eYFP was microinjected into one side of the RNm and the laser stimulation and recording electrodes targeted the same side of the RNm. (**G**) ChR2-eYFP was seen in RNm glutamate neurons (green) in VgluT2-Cre mice under high magnifications. (**H**) Representative RNm neuronal responses to repeated laser stimulation (2-ms light pulses of 473 nm). (**I**) Representative optical excitatory postsynaptic current (oEPSC) traces to light stimulation (200-ms pulse of 473 nm) when the membrane potential was clamped at −70 mV. (**J**) Average currents from all recorded cells reveal a peak current of −459.7 ± 89.80 pA and a steady-state current of −255.6 ± 65.76 pA (*n* = 11 cells). (**K**) RNm neuronal responses to different frequencies (1, 3, 10, and 20 Hz; 2-ms pulse duration) of light stimulation, displaying <5% loss of spike fidelity at 20 Hz (*n* = 8 cells). Scale bars, 250 and 50 μm (B and D) and 200, 50, and 10 μm (G) (also see fig. S3).

Next, we used triple-staining RNAscope in situ hybridization (ISH) assays to examine *Slc17a6* [for expressing vesicular glutamate transporter 2 (VgluT2), a glutamatergic neuronal marker in subcortical brain regions], *GAD1* (glutamic acid decarboxylase 1; a GABAergic neuronal marker), and *TH* (tyrosine hydroxylase; a DA neuronal marker) gene expression in the RN. We found that the dorsolateral RNp contains a mixture of small- to middle-sized glutamatergic and GABAergic neurons (~50:50) (Fig. 2, A to C, and fig. S3B). Conversely, the ventromedial RNm is composed of giant- to large-sized glutamatergic neurons (>75%, GAD^+^ versus VgluT2^+^, unpaired *t* test, *P* < 0.001) (Fig. 2, D and E, and fig. S3B). Both the RNp and anterior VTA (labeled by *TH* mRNA) are anatomically adjacent (Fig. 2B), while the RNm and the posterior VTA are adjacent at the caudal RN without significant overlap (Fig. 2D).

We then used optogenetic and electrophysiological methods to verify the glutamatergic phenotype of neurons in the RNm (Fig. 2F). To this end, AAV-DIO-ChR2-eYFP (AAV-ChR2-eYFP) was microinjected into the RNm unilaterally in VgluT2-IRES-Cre (VgluT2-Cre) mice to selectively express channelrhodopsin-2 (ChR2)–eYFP in glutamate neurons. We found high densities of eYFP-labeled glutamate neurons in the RNm (Fig. 2G). Whole-cell patch-clamp recordings showed that tissue taken from mice microinjected intra-RN with AAV-ChR2-eYFP displayed a reliable response to a 200-ms light pulse of 473 nm in RNm neurons (Fig. 2, H to J). RNm neurons also responded to different frequencies (1, 3, 10, and 20 Hz; 2 ms) of light stimulation with <5% loss of spike fidelity at 20 Hz (Fig. 2K), indicating that optically induced firing occurs in RNm glutamatergic neurons at physiologically relevant frequencies (*30*).

### Optical stimulation of RNm glutamate neurons is rewarding

**Fig. 3.**
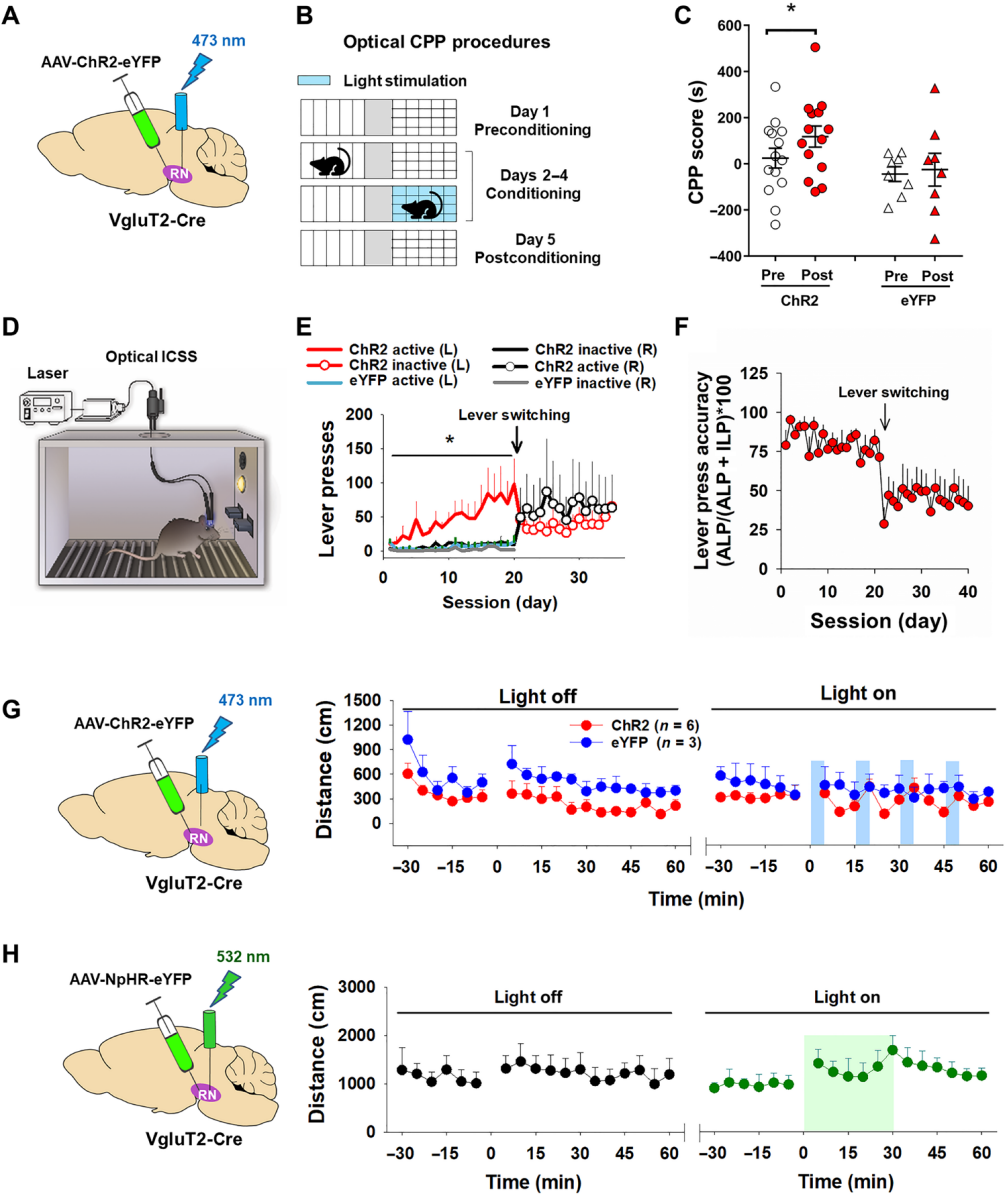
Behavioral responses to laser stimulation of RNm glutamate neurons in VgluT2-Cre mice. (**A**) Schematic diagram showing that AAV-eYFP or AAV-ChR2-eGFP was injected into bilateral RNm and the optrodes targeted to bilateral RNm. (**B**) Five days of optical CPP procedures. (**C**) Laser-paired conditioning produced a significant CPP response in mice with ChR2 expression in RNm glutamate neurons, but not in eYFP control mice. (**D**) Schematic diagram showing the oICSS system. (**E**) Laser stimulation of RNm glutamate neurons produced oICSS as assessed by progressively increased responding on the active lever over the inactive lever in the ChR2-expressing mice (*n* = 8), but not in the eYFP control mice (*n* = 4). After the active/inactive lever switching on day 21, animals rapidly learned to press the new active lever that was previously inactive. (**F**) Average lever press accuracy (%) (active lever responses/total number of active and inactive lever responses) indicating that animals discriminated the active lever from the inactive lever for brain-stimulation reward during the initial 20 days of oICSS training. (**G**) Optical stimulation of bilateral RNm glutamate neurons (with 5 min laser on followed by 10 min laser off) failed to alter open-field locomotion in VgluT2-Cre mice with AAV-ChR2 microinjection. (**H**) Optical inhibition of bilateral RNm glutamate neurons (with 30 min laser on followed by 30 min laser off) also failed to alter open-field locomotion in mice with AAV-NpHR-eYFP expression in RNm glutamate neurons (also see fig. S4).

To further explore the role of RNm glutamate neurons in exercise reward, we used CPP and optogenetic intracranial self-stimulation (oICSS) to determine whether stimulation of RNm glutamate neurons is rewarding. In this experiment, AAV-ChR2-eYFP was microinjected bilaterally into the RNm, with stimulation optrodes placed immediately above the RNm to target glutamatergic neuronal cell bodies (Fig. 3A) in VgluT2-Cre mice. After recovery from surgery, the animals were first placed in CPP chambers in the alternating absence or presence of laser stimulation (20 Hz, 10-ms duration, 473-nm wavelength, 10 mW, 15 min/day) (Fig. 3B). After 3 days of laser-paired conditioning, mice transduced with intra-RNm AAV-ChR2-eYFP virus displayed significant CPP (paired *t* test, *P* < 0.05; *n* = 13; Fig. 3C, left), while those that received the control virus (AAV-eYFP) showed neither CPP nor conditioned place avoidance (paired *t* test, *P* = 0.82; *n* = 8; Fig. 3C, right).

Next, these mice were placed in operant oICSS chambers and trained to make a behavioral response for optical stimulation of glutamatergic neurons in the RNm. With each active lever press, a train of laser pulses (20 Hz, 40 pulses, 473-nm wavelength, 10-ms duration, 10 mW) was delivered into the RNm (Fig. 3D) during daily 60-min testing sessions. Responses on the inactive lever were not paired with laser stimulation and had no consequence. Figure 3E shows that control mice with an intra-RNm microinjection of the control virus [adeno-associated virus (AAV)–eYFP] did not show significant lever responses to laser stimulation. However, the ChR2-expressing mice exhibited gradual discrimination between the active and inactive levers and progressive increases in active lever responding during the 20 days of oICSS availability. A two-way RM ANOVA revealed a significant difference between active and inactive lever responses from day 1 to day 20 [ChR2 active (L) versus ChR2 inactive (R); *F*_1,10_ = 6.07, *P* < 0.05; Fig. 3E]. After reversal of active versus inactive levers, animals rapidly learned to press the new active lever (Fig. 3E), suggesting that laser stimulation of RNm glutamate neurons is rewarding. Figure 3F shows the average lever press accuracy (% active lever responses over the total number of active and inactive lever responses). We found that mice were able to discriminate the active lever from the inactive lever in response to laser stimulation during the initial 20 days of oICSS training.

We also observed open-field locomotor responses to laser stimulation. Bilateral stimulation of RNm glutamate neurons (473-nm wavelength, 20 Hz, 10-ms duration, 10 mW, 5 min on and 10 min off) failed to alter locomotor activity in VgluT2-Cre mice that had received intra-RNm AAV-ChR2-eYFP microinjections (two-way RM ANOVA, laser off: ChR2 versus eYFP, *F*_1,7_ = 5.52, *P* = 0.051, *n* = 8; laser on: ChR2 versus eYFP, *F*_1,7_ = 1.29, *P* > 0.05, *n* = 8; Fig. 3G). Similarly, optogenetic inhibition of RNm glutamate neurons (532 nm, 10 mW per side, constant, for 30 min) failed to alter open-field locomotion in VgluT2-Cre mice that had received intra-RNm AAV-NpHR-eYFP microinjections (two-way RM ANOVA, laser off versus laser on: *F*_1,6_ = 0.0044, *P* > 0.05, *n* = 7; Fig. 3H).

### RNm glutamate neurons project to the VTA

We next explored the neural mechanisms underlying RNm-mediated reward. To address this question, we focused on RNm glutamatergic neuronal projections and inputs. AAV-ChR2-eYFP was injected into the RNm of VgluT2-Cre mice unilaterally (fig. S5A) or bilaterally (fig. S5B) to examine eYFP-labeled glutamate neurons and their fiber projections. We found that RNm glutamate neurons project bilaterally to the VTA (fig. S5, B and C), particularly to the contralateral VTA (fig. S5, A and B). We also examined TH^+^ DA neuronal projections and found that VTA DA neurons project to the RNm (fig. S5D), suggesting the presence of reciprocal connections between the RNm and VTA.

The retrograde tracer Fluoro-Gold (FG) was used to examine afferents to the RNm. In this experiment, FG was microinjected into the RNm (fig. S6A) and FG staining was imaged in slices. FG staining was detected in several brain areas, including the ipsilateral substantia nigra pars reticulata (SNr), cortex, contralateral RNm, bilateral superior colliculus, and cerebellum (fig. S6, A to C), suggesting that the RNm is a critical midbrain motor structure that integrates input from both cortical and subcortical brain regions (*31*).

**Fig. 4.**
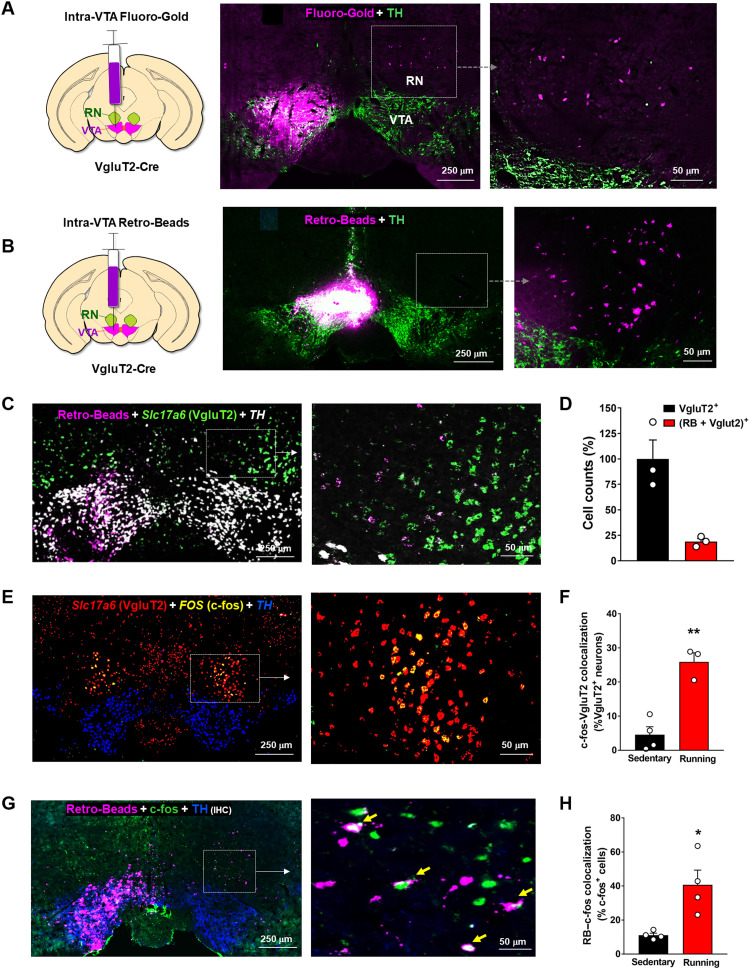
Identification of the RN→VTA glutamate projections. (**A**) Schematic diagram showing that Fluoro-Gold was injected unilaterally into the VTA of a VgluT2-Cre mouse. Fluoro-Gold-labeled neurons were detected in the contralateral RN. (**B**) The retrograde tracer Red-Beads (RB) was injected unilaterally into the VTA, and RB-labeled neurons were found in the contralateral RNm. (**C** and **D**) Representative fluorescent RB tracing and RNAscope assays, illustrating that RB was colocalized with VgluT2 mRNA (*Slc17a6*) in ~20% VgluT2^+^ glutamate neurons in the contralateral RNm. (**E** and **F**) Triple-staining RNAscope assays, indicating that wheel-running increased *FOS* (c-fos) expression in ~25% VgluT2^+^ glutamate neurons in bilateral RNs. (**G**) Representative images showing that red RBs were injected into one side of the VTA to label exercise-induced c-fos expression in the RN-VTA projection neurons (marked by arrows) in the contralateral RNm. (**H**) Quantitative cell counting data, indicating RB and c-fos colocalization in ~40% c-fos–expressing neurons in mice after 10 days of wheel-running. Scale bars, 250 and 50 μm (A to C, E, and G). *P < 0.05 and **P < 0.01, compared to the sedentary control group (also see figs. S5 and S6).

To further determine whether these RN→VTA glutamatergic neurons directly project onto VTA neurons or merely the nerve fibers that travel through the VTA, FG or RetroBeads (RBs; another red fluorescent retrograde tracer) were locally injected on one side of the VTA (Fig. 4, A and B). We detected FG- or RB-labeled neurons in the contralateral RNm (Fig. 4, A and B), suggesting that a subpopulation of RNm glutamate neurons project directly to the VTA.

To ascertain the phenotype of the RB-labeled neurons, we used retrograde RB tracing techniques combined with RNAscope ISH assays. We found that RB and *Slc17a6* mRNA were colocalized in ~20% of VgluT2^+^ glutamate RNm neurons (Fig. 4, C and D), suggesting that a small population of RNm glutamate neurons directly project to the VTA.

Next, we examined whether wheel-running activates RNm glutamate neurons. We found that wheel-running significantly increased *FOS* mRNA expression in ~25% of VgluT2-expressing glutamate neurons (Fig. 4, E and F). Notably, this c-fos response occurred exclusively in VgluT2^+^ neurons but not in VgluT2^−^ (e.g., nonglutamate) neurons (Fig. 4E). This increase in glutamatergic c-fos expression was significantly higher in wheel-running mice than in wheel-locked controls (sedentary versus running, unpaired *t* test, *P* < 0.01; Fig. 4F). We also examined whether physical exercise can activate the RN→VTA projection pathway. To this end, RBs were microinjected into one side of the VTA to label the RNm→VTA projection neurons. We found that RBs were colocalized with *FOS* in ~40% of c-fos–expressing neurons (Fig. 4, G and H) (sedentary versus running, unpaired *t* test, *P* < 0.05), suggesting that wheel-running exercise does activate a subset of RN→VTA glutamatergic projection neurons.

### RNm glutamate projection neurons synapse onto VTA DA neurons

**Fig. 5.**
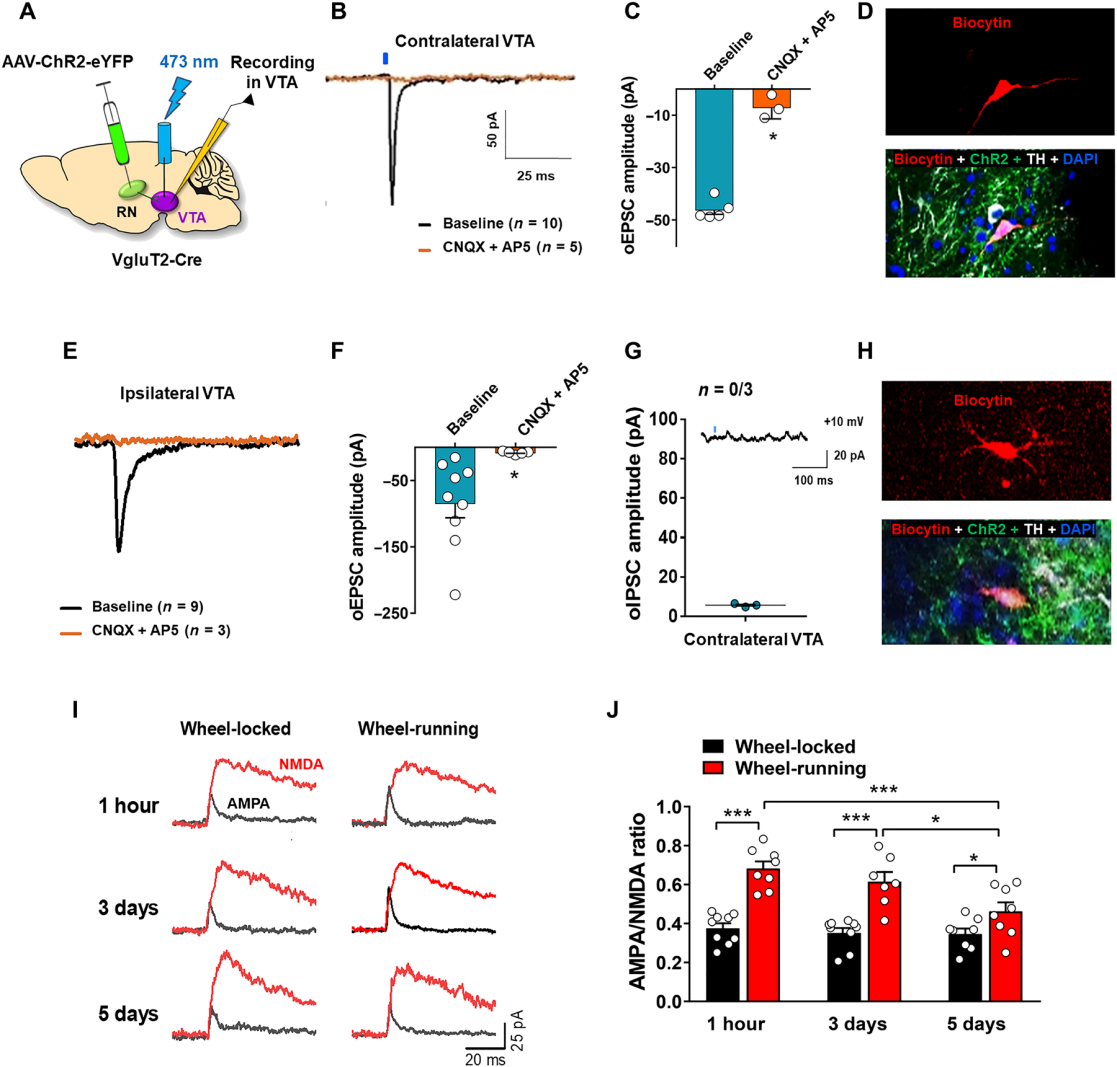
Functional characterization of the RN-VTA glutamate projection neurons. (**A**) Schematic diagram of an electrophysiological experiment, indicating that AAV-ChR2-eYFP was injected unilaterally into the RNm, while the stimulation and recording optrodes targeted contralateral VTA DA neurons. (**B** and **C**) Representative oEPSC trace and the mean oEPSC response of VTA DA neurons to laser stimulation of glutamate terminals in the VTA projecting from the contralateral RNm. Application of CNQX and AP5 blocked the oEPSCs. (**D**) Representative images showing that a recorded VTA neuron is a TH^+^ DA neuron, as labeled by biocytin (red) and TH (white) after the completion of the recording. (**E** and **F**) Representative oEPSC trace and the mean oEPSC response of VTA DA neurons to light stimulation of ipsilateral glutamate terminals in the VTA, which was also blocked by CNQX + AP5. (**G**) Optical stimulation of glutamate terminals in the VTA did not evoke optical inhibitory postsynaptic currents (oIPSCs) in contralateral VTA DA neurons when the membrane potential was held at +10 mV (*n* = 3, *P* > 0.05). (**H**) Another set of representative images showing that a recorded VTA neuron is a TH^+^ DA neuron, as labeled by biocytin after the completion of the recording. (**I**) Representative AMPAR- and NMDAR-EPSCs recorded from RNm projecting DA neurons in the contralateral VTA of the midbrain slices prepared from wheel-locked and wheel-running mice 1 hour, 3 days, or 5 days after the last training. (**J**) Compared with time-matched wheel-locked controls, wheel-running produced an increase in AMPAR/NMDAR ratio, which lasted for 5 days, suggesting long-lasting synaptic potentiation. *P < 0.05 and ***P < 0.001.

To determine whether the RN→VTA glutamatergic projection neurons form functional synapses with VTA DA neurons, we injected AAV-ChR2-eYFP into the RNm unilaterally in VgluT2-Cre mice and then optically stimulated VTA glutamate terminals in brain slices (Fig. 5A). Stimulation of RNm glutamate terminals produced optical excitatory postsynaptic currents (oEPSCs) in contralateral VTA DA neurons (Fig. 5, B and C). The average latency of oEPSCs was 1.51 ± 0.21 ms, suggesting monosynaptic connections. Application of the combination of CNQX [an AMPA receptor (AMPAR) antagonist] and AP5 [an *N*-methyl-d-aspartate receptor (NMDAR) antagonist] completely abolished the evoked currents (baseline versus CNQX + AP5, unpaired *t* test, *P* < 0.001; Fig. 5C), indicating that evoked oEPSCs in VTA DA neurons are glutamate dependent. After patch recording, biocytin was delivered into the recorded neurons, followed by labeling the cells with anti-TH antibody. The results indicated that these cells were TH^+^ DA neurons (Fig. 5D).

Optical stimulation of ipsilateral glutamate terminals also evoked oEPSCs (Fig. 5E), which were also CNQX sensitive (Baseline versus CNQX + AP5, unpaired *t* test, *P* < 0.05; Fig. 5F), indicating glutamate-mediated currents. When recording inhibitory postsynaptic currents (IPSCs) in VTA DA neurons in the same slices, we found that optical stimulation of RNm glutamate terminals failed to evoke optical IPSCs (oIPSCs) in VTA DA neurons (Fig. 5G), suggesting that these neurons do not co-release GABA or evoke feed-forward inhibition through excitation of GABAergic interneurons. These electrophysiological findings indicate that the RNm glutamate projection neurons are capable of driving DA neuron activity in the VTA.

### Wheel-running enhances glutamate transmission from the RNm to VTA DA neurons

We then examined whether wheel-running alters the RNm-VTA glutamate transmission onto VTA DA neurons. For this purpose, AAV-ChR2-eYFP was microinjected into the RNm unilaterally in VgluT2-Cre mice to express ChR2-eYFP in RNm glutamate neurons and their projection terminals. Four weeks following surgery (to allow ChR2-eYFP expression), the animals were trained to wheel-run for 10 days as shown in Fig. 1A. Optically evoked EPSCs were recorded from contralateral DA neurons 1 hour, 3 days, and 5 days after the final wheel-running training. Figure 5I shows representative AMPAR-mediated currents and NMDAR-mediated currents at three different time points from the last wheel-running training. Figure 5J shows the AMPAR/NMDAR ratio, indicating that wheel-running significantly increased glutamate transmission from the RNm to VTA DA neurons, an effect that lasted for at least 5 days. A two-way ANOVA revealed a significant wheel-running treatment main effect (*F*_1,43_ = 63.73, *P* < 0.001), time main effect (*F*_2,43_ = 6.50, *P* < 0.01), and wheel-running × time interaction (*F*_2,43_ = 4.06, *P* < 0.05). Post hoc individual group comparisons showed significant differences between the wheel-running and wheel-locked groups measured at 1 hour, 3 days, and 5 days from the last session.

### Optical stimulation of the RN→VTA glutamate terminals is rewarding

**Fig. 6.**
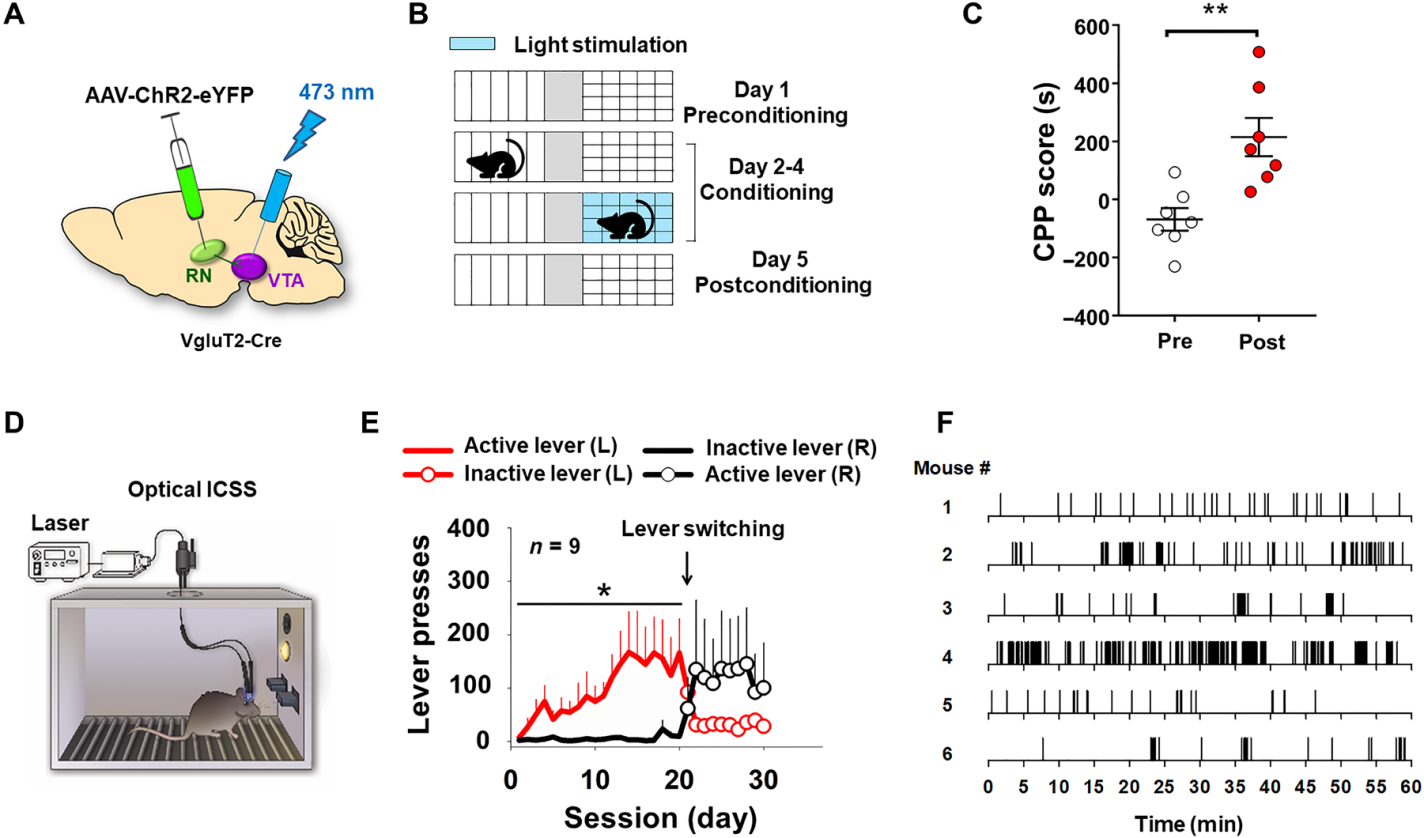
Optical stimulation of RNm glutamate terminals in the VTA is rewarding in VgluT2-Cre mice. (**A**) Schematic diagram showing that AAV-ChR2-eYFP was injected to bilateral RNm and the optrodes targeted the bilateral VTA. (**B**) Five days of optical CPP procedures. (**C**) Laser-paired conditioning produced a significant CPP response (e.g., mice spent more time in the laser stimulation–paired chamber). (**D**) Mouse oICSS chamber with the optogenetic device installed. (**E**) Laser stimulation of RNm glutamate terminals in the VTA produced oICSS responding on the active lever in ChR2-expressing mice (*n* = 9). After the active/inactive lever switching on day 21, animals rapidly learned to press the new active lever that was inactive previously. (**F**) Representative active lever responding records from six ChR2-expressing animals on day 20, illustrating robust oICSS behavior to the laser stimulation of the RN-VTA glutamate terminals (also see fig. S7). *P < 0.05, **P < 0.01, compared to Inactive lever responding (E) or Pre-conditioning (C).

We next evaluated whether selective stimulation of RNm→VTA glutamatergic terminals is rewarding. To this end, we used the same optical CPP and oICSS techniques described above, in which AAV-ChR2-eYFP was injected bilaterally into the RNm, with optrodes targeted on RNm glutamate terminals in the VTA (Fig. 6A) of VgluT2-Cre mice. Mice with ChR2 expression in RNm glutamate neurons displayed significant CPP, i.e., increased time spent in the laser-paired compartment (Fig. 6C) (paired *t* test, *P* < 0.001). Given that wheel-running produced a long-lasting increase in the RN-VTA glutamate transmission, we examined whether optical stimulation of the RN-VTA glutamate pathway–induced CPP is long-lasting. Stimulation of the glutamate terminals in the VTA produced long-lasting CPP—up to 3 days from the last laser stimulation conditioning (fig. S4).

To confirm the above finding, we used oICSS procedures to observe whether contingent stimulation of the RNm→VTA glutamate terminals can maintain operant behavior. In this experiment, when the animals pressed the active lever, a train of laser pulses (20 Hz, 40 pulses, 473-nm wavelength, 10-ms duration, 10 mW) was delivered to the VTA (Fig. 6D) during daily 60-min testing sessions. ChR2-expressing mice exhibited rapid discrimination between the active and inactive levers [active (L) versus inactive (R), two-way RM ANOVA, *F*_1,16_ = 4.66, *P* < 0.05; Fig. 6E] and progressive increases in active lever responding during 20 days of oICSS availability. Similarly, following active/inactive lever reversal (Fig. 6E), animals rapidly learned to press the new active lever. Figure 6F shows representative active lever responding of six representative animals on day 20 (i.e., before lever switching), illustrating stable robust oICSS behavior maintained by optical activation of RN-VTA glutamate terminals.

### Optical stimulation of VTA DA neurons is rewarding

To confirm whether VTA DA neurons are the downstream target of the RN-VTA glutamate projections, we used the same optogenetic approaches to directly activate VTA DA neurons in DAT-Cre mice (fig. S7A). We observed robust locomotor responses to laser stimulation in open-field locomotion (fig. S7, B and C) (one-way RM ANOVA, *F*_23,138_ = 5.59, *P* < 0.001; fig. S7C). We also observed laser-stimulated CPP (*F*_1,6_ = 51.14, *P* < 0.001; fig. S7D) and oICSS (fig. S7, E and F), which is consistent with our previous report that optogenetic stimulation of VTA DA neurons is rewarding as assessed by optical real-time place preference and oICSS (*32*). Together, these findings from both the CPP and oICSS paradigms indicate that activation of either the cell bodies of RNm glutamate neurons (Fig. 3), their terminals in the VTA (Fig. 5), or the downstream VTA DA neurons (fig. S7) is rewarding and suggest that the runner’s high might be mediated at least in part by activation of this newly identified RN→VTA glutamate pathway.

### Inhibition of the RN-VTA glutamate pathway attenuates exercise reward

**Fig. 7.**
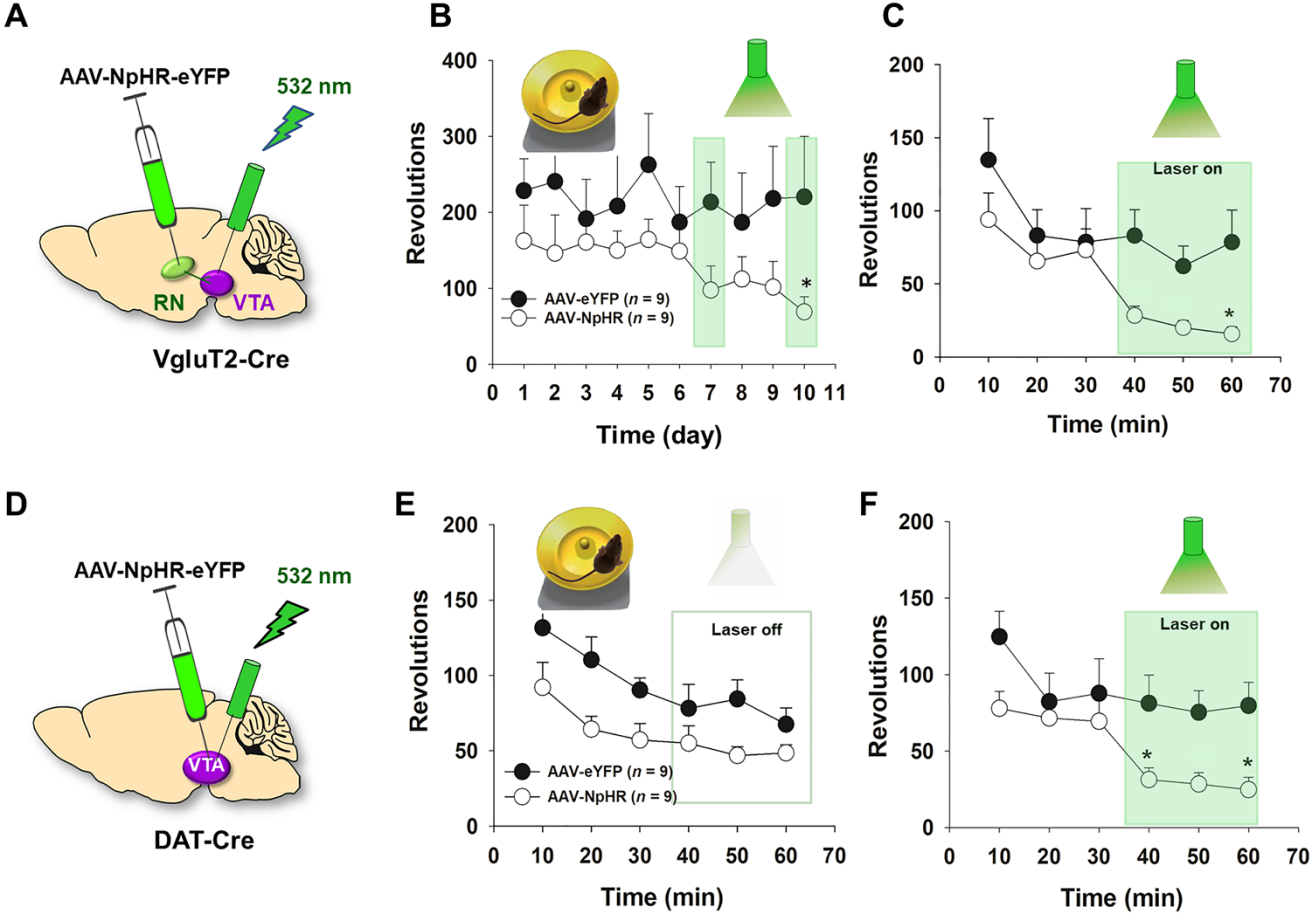
Optical inhibition of the RN-VTA glutamate pathway attenuates wheel-running behavior. (**A**) Schematic diagram of the general procedures, in which AAV-NpHR-eYFP was injected into bilateral RNm and the stimulation optrodes targeted the bilateral VTA. (**B**) Time course of wheel-running over 10 consecutive days of daily 60-min sessions, showing the second 30-min data (number of revolutions) in the presence or absence of green laser (532 nm) stimulation. (**C**) Time courses of wheel-running within daily 60-min sessions on the last laser test day, illustrating that optical inhibition of glutamate release from RNm glutamate terminals in the VTA inhibited wheel-running behavior. (**D**) Schematic diagram of the general procedures, in which AAV-NpHR-eYFP was injected into bilateral VTA and the stimulation optrodes also targeted the bilateral VTA. (**E** and **F**) Time courses of wheel-running within daily 60-min sessions in mice on the last laser test day, illustrating that optical inhibition of VTA DA neurons (via NpHR) robustly decreased wheel-running behavior. *P < 0.05, compared to the eYFP control group.

To further test the above exercise reward hypothesis, we examined whether optogenetic inhibition of RNm-VTA projection glutamate neurons or VTA DA neurons can block wheel-running behavior. To this end, AAV-NpHR-eYFP or the control virus (AAV-eYFP) was injected bilaterally into the RNm of VgluT2-Cre mice and optrodes were targeted to the VTA (Fig. 7A). Each animal ran for 60 min/day in the test room for 10 consecutive days with wheel-running available in their home cages (during the dark cycle). Once the running behavior was stabilized for at least 2 to 3 days, each animal received green laser stimulation (532 nm, 10 mW per side, constant on for 30 min) during the last 30 min of wheel-running access to inhibit glutamate release from the RNm-VTA glutamate terminals. Figure 7B shows the time courses of wheel-running during the second 30-min period over 10 daily sessions, illustrating that optical inhibition of glutamate release from the RN-VTA glutamate terminals significantly inhibited wheel-running behavior as compared to the baseline or the control virus group (eYFP group revolutions on test days versus NpHR group revolutions on test days, two-way RM ANOVA, laser treatment main effect, *F*_1,16_ = 5.88, *P* < 0.05). Figure 7C shows the time courses of wheel-running within a daily 60-min session, indicating that laser stimulation inhibited wheel-running. A two-way RM ANOVA revealed a significant laser treatment main effect (*F*_1,16_ = 5.88, *P* < 0.05; Fig. 7C) during the 30 min with laser manipulation. Post hoc individual group comparisons revealed a significant reduction (*P* < 0.05) in wheel-running in the NpHR treatment group compared to the AAV-eYFP control group at 60 min.

We then used the same experimental design to inhibit VTA DA neurons (Fig. 7D) by microinjecting AAV-NpHR-eYFP or the control virus into the VTA of DAT-Cre mice. We found that optical inhibition of VTA DA neurons also inhibited wheel-running (Fig. 7, E and F). A two-way RM ANOVA revealed a significant laser treatment main effect (*F*_1,12_ = 8.15, *P* < 0.05, Fig. 7F). Post hoc individual group comparisons revealed a significant difference in wheel-running between the eYFP and NpHR groups at 40 and 60 min (Fig. 7F). These findings suggest that wheel-running is RN-VTA glutamate dependent and DA dependent. We infer that activation of the RNm-VTA glutamate pathway, at least in part, underlies the runner’s high and exercise reward. This is consistent with the widely accepted view that a DA-dependent mechanism may underlie both natural (food, sex, and exercise) reward and drug reward (*33*, *34*).

### Wheel-running attenuates cocaine self-administration and cocaine intake

As stated above, physical exercise such as wheel-running can attenuate the effects of drugs of abuse in both humans and experimental animals (*35*–*37*). However, the neural mechanisms through which exercise attenuates drug intake are poorly understood. Given the above findings, we hypothesized that wheel-running may inhibit cocaine self-administration by activation of RNm-VTA glutamate circuitry.

**Fig. 8.**
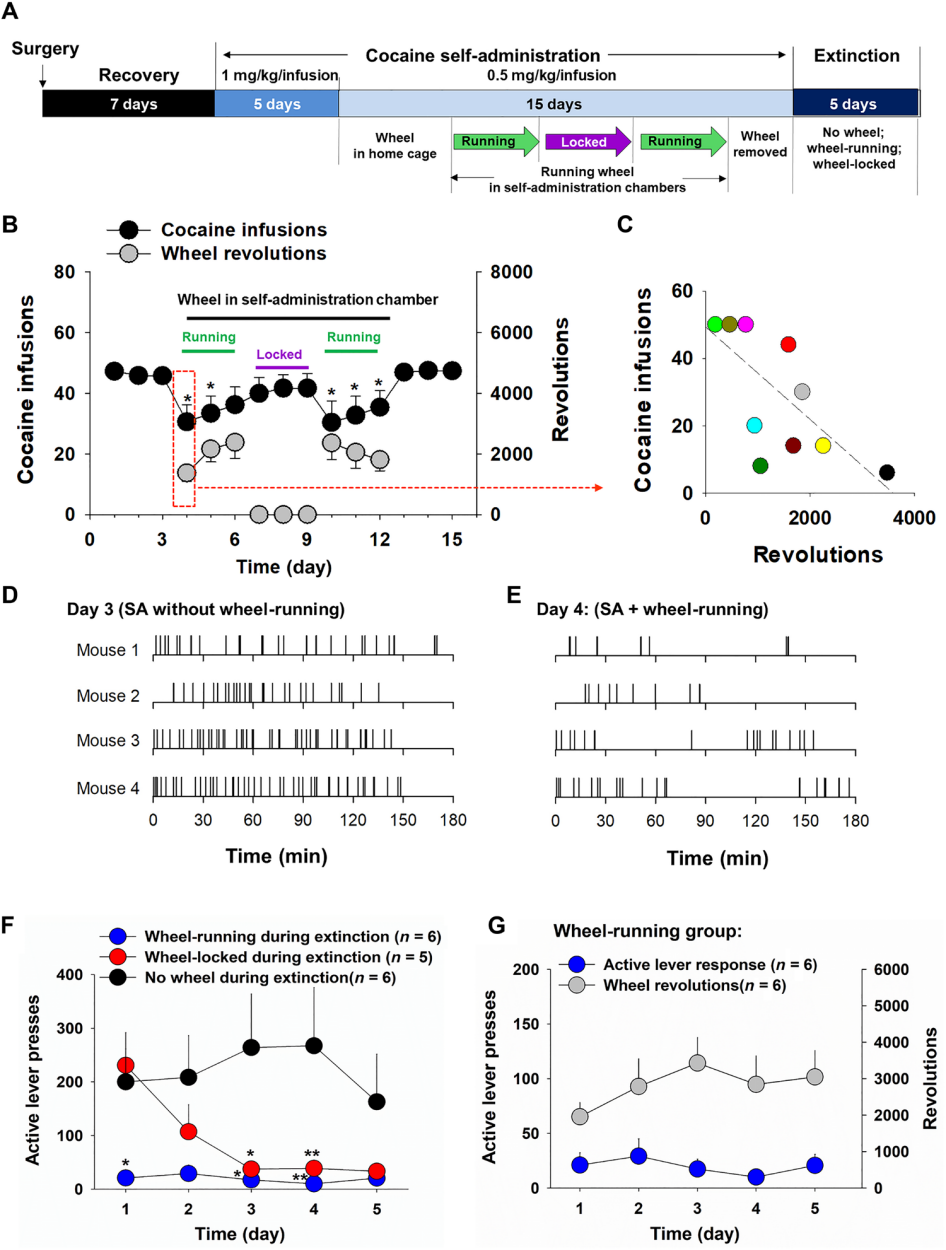
Effects of wheel-running on intravenous cocaine self-administration and cocaine seeking during extinction in mice. (**A**) Timeline of the experimental procedures. (**B**) Results of cocaine versus wheel-running choice tests, illustrating that mice took less cocaine in the presence of running wheel during the daily 3-hour self-administration sessions under fixed ratio 1 (FR1) reinforcement. (**C**) Correlation between cocaine infusions and wheel-running performed on the data observed on day 4 [shown in (B)], indicating that mice ran more and took less cocaine during the cocaine versus wheel-running choice test. (**D** and **E**) Representative cocaine self-administration (SA) records indicating a significant reduction in cocaine self-administration (infusions) with an extinction-like pattern of drug taking in the presence of wheel-running on day 4 (E) compared to that in the absence of active wheel-running on day 3 (D). (**F**) Extinction responding (e.g., active lever presses) in three groups of mice, indicating that mice with active wheel-running (blue circles) displayed very low levels of drug-seeking behavior compared to mice without wheel-running experience before and during cocaine self-administration and extinction (black circles). Unexpectedly, mice with wheel-running experience during cocaine self-administration but without active wheel-running (wheel-locked) during extinction (red circles) also displayed rapid extinction (i.e., less cocaine-seeking over time). (**G**) Wheel-running versus cocaine-seeking (i.e., active lever presses) performance in mice with active wheel-running during five consecutive days of daily extinction. *P < 0.05 and **P < 0.01, compared to baseline or no wheel access control group.

To test this hypothesis, we first confirmed that wheel-running exercise inhibits cocaine self-administration and cocaine choice. In this experiment, male mice were allowed to acquire cocaine self-administration until stable self-administration was achieved, which was defined as (i) at least 20 infusions per 3-hour session, (ii) less than 20% variability in daily cocaine infusions across two consecutive sessions, and (iii) an active/inactive lever press ratio exceeding 2:1. Before the cocaine self-administration and choice tests, the animals received 3 days of wheel-running experience in their home cages, following the completion of daily cocaine self-administration. The wheel-running devices were then moved to the self-administration chambers, in which animals could choose to lever press for cocaine or wheel-running exercise under various conditions (Fig. 8A). We found that the mice exhibited both significant wheel-running and cocaine self-administration. However, the total number of cocaine self-administration infusions was significantly decreased in the presence of active wheel-running as compared to baseline (Fig. 8B). A one-way RM ANOVA showed a significant treatment main effect (*F*_14,140_ = 3.94, *P* < 0.001). Post hoc Turkey’s tests revealed a significant reduction in cocaine self-administration in the presence of active wheel-running on days 4 and 5 and days 10 to 12 (*P* < 0.05) compared to baseline responding. To determine whether the presence of the running wheel device may act as a novel object distracting the animals from cocaine self-administration, the running wheels were either locked during the following 3 days (days 7 to 9) or taken away from the self-administration chambers on the last 3 days of testing (days 13 to 15) (Fig. 8A). In both cases, cocaine self-administration returned to baseline levels (Fig. 8B). When the running wheels were unlocked again on days 10 to 12, self-administration decreased again. Figure 8C shows an inverse correlation between wheel-running and cocaine self-administration infusions based on the data from the first wheel-running test day (day 4), illustrating that when mice ran more, they took less cocaine. The animals displayed atypical extinction-like patterns—initial drug self-administration followed by cessation of drug intake or irregular cocaine-taking behavior (Fig. 8, D and E), suggesting a decreased interest in cocaine when wheel-running was available.

Next, subjects were placed on an extinction schedule, during which the running wheels were either locked or unlocked, while active lever presses had no consequence (i.e., the cue light and cocaine-filled pumps were turned off). When compared to the cocaine-seeking behavior in control mice with no wheel-running experience, mice with prior exposure to the running wheels exhibited much lower active lever responding (cocaine-seeking) both when the wheels were active (unlocked) or inactive (locked) (Fig. 8F). A two-way RM ANOVA revealed a significant treatment main effect (*F*_2,14_ = 6.42, *P* < 0.05; Fig. 8F). Post hoc individual group comparisons revealed a significant difference between the no wheel access and the wheel-running groups. The mice with unlocked wheels displayed robust wheel-running behavior but no cocaine-seeking behavior (Fig. 8G). Thus, wheel-running or previous wheel-running experience significantly reduces motivation for cocaine seeking during extinction.

**Fig. 9.**
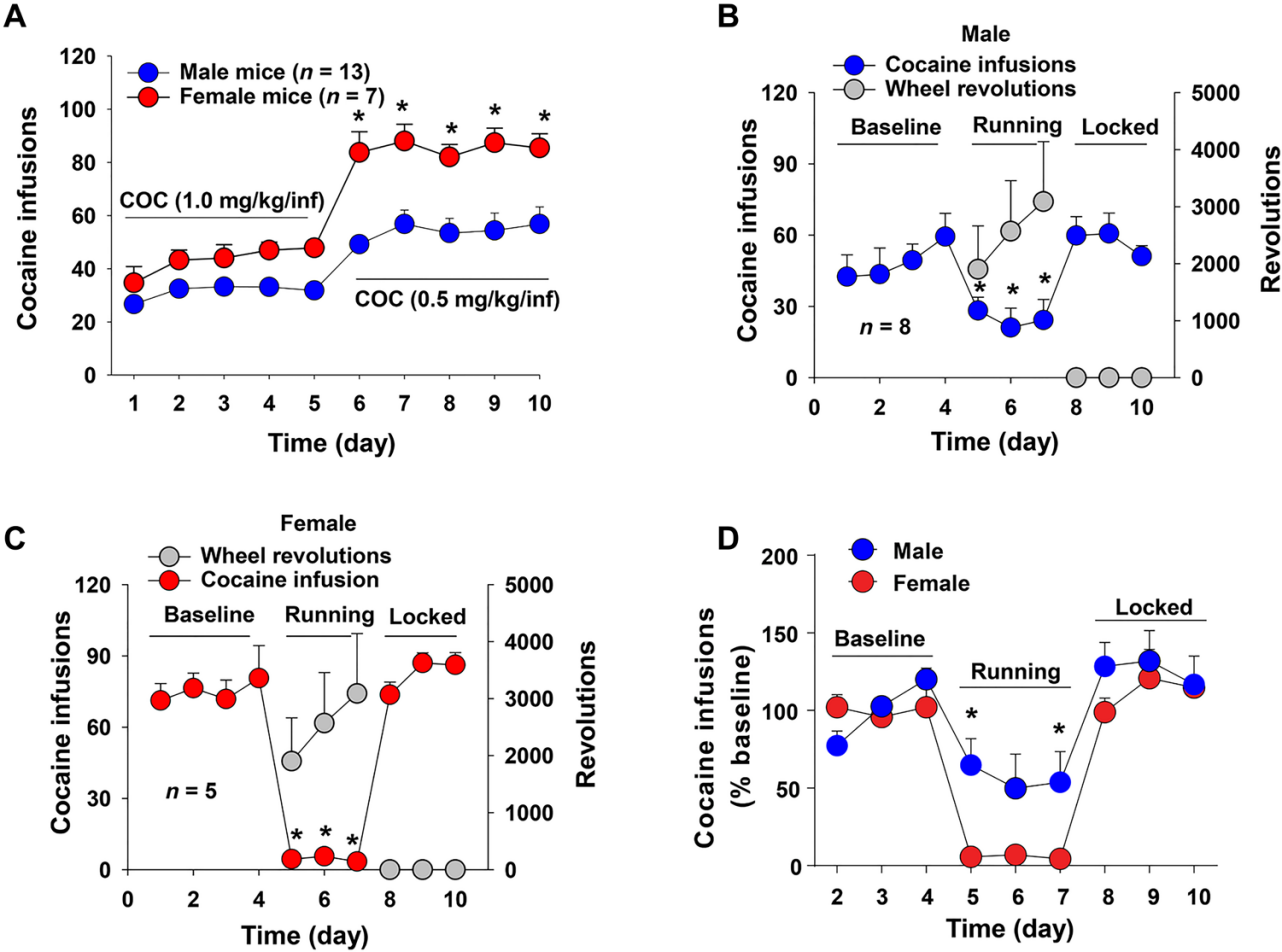
Effects of wheel-running on cocaine self-administration in male and female mice. (**A**) Sexual difference in cocaine (COC) self-administration maintained by two doses of cocaine, indicating that females took more cocaine than males under the same experimental conditions. (**B**) Cocaine self-administration in males in the presence of wheel-running choice versus when the wheel was locked. (**C**) Cocaine self-administration in females in the presence of wheel-running choice versus when the wheel was locked. (**D**) Average % changes in cocaine self-administration in the presence of unlocked versus locked wheels. Wheel-running inhibited cocaine self-administration in both males and females, but it was more effective in reducing cocaine self-administration in females. *P < 0.05, compared to baseline.

We also examined sex differences in cocaine self-administration and behavioral responsivity to wheel-running. Figure 9A shows that female mice self-administered more cocaine than males, particularly at the lower dose (0.5 mg/kg per infusion). A two-way RM ANOVA over time revealed a significant sex main effect (*F*_1,18_ = 16.52, *P* < 0.05), time main effect (*F*_9,162_ = 52.2, *P* < 0.05), and sex × time interaction (*F*_9,162_ = 4.82, *P* < 0.05). Post hoc testing for multiple group comparisons indicated significant sex differences in the number of cocaine infusions (0.5 mg/kg per infusion) between males and females (Fig. 9A). The presence of wheel-running produced a significant reduction in cocaine self-administration in both males (*F*_6,42_ = 5.58, *P* < 0.05; Fig. 9B) and females (*F*_6,24_ = 33.01, *P* < 0.05; Fig. 9C), but a more robust reduction in cocaine self-administration was observed in females (Fig. 9D) (sex × time interaction, *F*_8,88_ = 2.15, *P* < 0.05).

### Stimulation of RNm glutamate neurons decreases cocaine self-administration

We then asked whether direct stimulation of the RN-VTA glutamate pathway can mimic wheel-running activity. To this end, we used a cocaine self-administration paradigm combined with optogenetic strategies to manipulate either cell bodies of RNm glutamate neurons, their terminals in the VTA in VgluT2-Cre mice, or their downstream VTA DA neurons in DAT-Cre mice. Mice were first allowed to acquire cocaine self-administration and habituated to the tethered fiber-optic cables until the self-administration behavior stabilized. Optical stimulation was delivered intracranially during cocaine self-administration (fig. S8, A to C). We first optimized the laser parameters that alter cocaine self-administration without producing unwanted side effects (fig. S8D). We found that 473-nm blue light (at 20 Hz, 10-ms pulse, 10 mW, 5 min laser on, 10 min laser off) was more effective than other parameters at inhibiting cocaine self-administration (fig. S8, D to F). Inhibition of cocaine self-administration caused by stimulation of bilateral RNm glutamate neurons was long-lasting (up to 3 days) after the termination of stimulation (fig. S8, E and F, right), which is consistent with our findings with optical CPP (fig. S4).

**Fig. 10.**
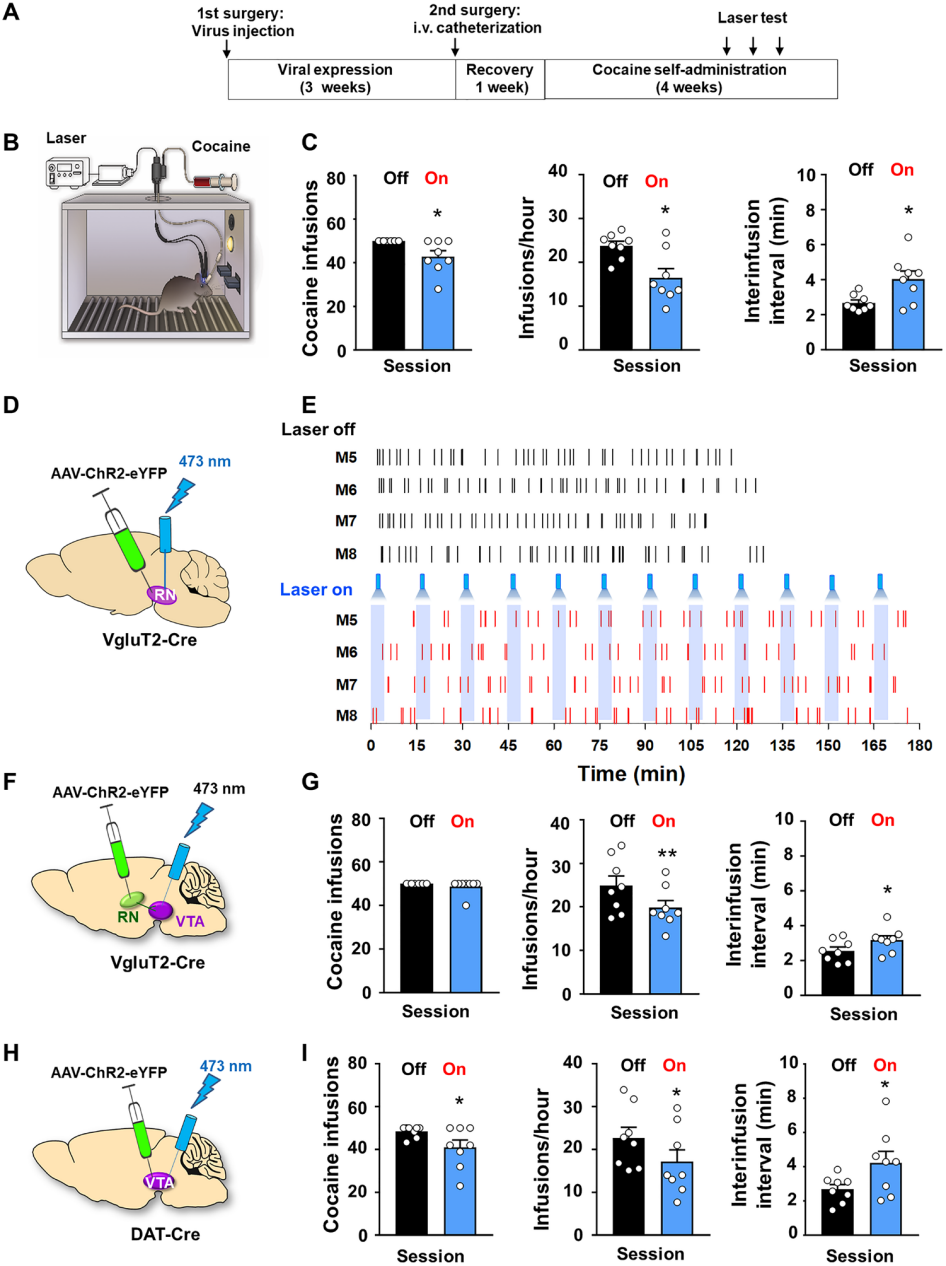
Effects of optical stimulation of the RNm-VTA glutamate pathway on cocaine self-administration. (**A**) Timeline of the general experimental procedures. (**B**) Schematic diagram showing mouse cocaine self-administration combined with optical brain stimulation. (**C**) Optogenetic stimulation of RNm glutamate neurons inhibited cocaine self-administration manifested as a significant reduction in total number of cocaine infusions and in the rate of cocaine infusions with a significant increase in interinfusion intervals. (**D**) Schematic diagram showing that AAVs were microinjected into the bilateral RNm and optrodes targeted the RNm. (**E**) Representative cocaine self-administration records from four animals, illustrating that laser stimulation (473 nm, 10 mW, 5-min laser on followed by 10-min laser off) decreased the rate of cocaine self-administration. The mice still displayed a regular and evenly distributed pattern of cocaine self-administration but with increased interinfusion intervals and reduced cocaine self-administration rates in the presence of laser stimulation. (**F** and **G**) Optical stimulation of the RNm glutamate terminals in the VTA also inhibited cocaine self-administration (*n* = 7). (**H** and **I**) Optical stimulation of VTA DA neurons significantly inhibited cocaine self-administration in a manner similar to stimulation of RNm glutamate neurons (cell bodies) (C) or their projection terminals in the VTA (G). *P < 0.05 and **P < 0.01, as compared to the laser off session (also see figs. S8 to S12).

We then observed the effects of RNm laser stimulation on cocaine self-administration (Fig. 10, A, B, and D). We found that stimulation of bilateral RNm glutamate neurons lowered cocaine infusions (paired *t* test, *P* < 0.05, *n* = 8; Fig. 10C) and the rate of cocaine self-administration (paired *t* test, *P* < 0.05; Fig. 10C) and increased the interinfusion intervals (paired *t* test, *P* < 0.05; Fig. 10C). Figure 10E shows the pattern of cocaine self-administration, illustrating that animals displayed stable and evenly distributed cocaine self-administration with significantly increased interinfusion intervals.

To determine whether the reduction in cocaine self-administration after RNm laser stimulation might be due to a nonspecific lighting effect, the same light stimulation was delivered into the RNm of the control mice with the AAV-eYFP microinjection in which no light-sensitive ChR2 was expressed in RNm glutamate neurons. This laser stimulation did not produce any effect on cocaine self-administration (fig. S9, A to C). In addition, we injected AAV-NpHR-eYFP into the RNm in VgluT2-Cre mice (fig. S9D). We found that optical inhibition of RNm glutamate neurons (532 nm, 10 mW per side, 30 min on, 30 min off) produced a mild increase in cocaine infusion rate without significant alteration in total number of cocaine infusions or averaged interinfusion intervals (fig. S9, E and F). These findings suggest that RNm glutamate neurons may tonically regulate neural circuits controlling brain reward function under normal physiological conditions.

### Stimulation of the RN→VTA glutamate terminals inhibits cocaine self-administration

Next, we examined whether optical stimulation of RNm-VTA glutamate terminals alters cocaine self-administration in a manner similar to stimulation of RNm glutamate neuronal cell bodies. As stated above, some RNm glutamate neurons project directly to the VTA and synapse onto DA neurons. Therefore, we hypothesized that stimulation of RNm glutamate terminals in the VTA should also inhibit cocaine self-administration. As expected, optogenetic stimulation of RN-VTA glutamate terminals within the VTA produced a significant reduction in cocaine self-administration in a manner similar to stimulation of RNm glutamate neurons (Fig. 10, F and G). Paired *t* tests failed to reveal a significant change in total number of cocaine infusions (paired *t* test, *P* > 0.05; Fig. 10G, left) but showed a significant reduction in cocaine infusion rate (paired *t* test, *P* < 0.05; Fig. 10G, middle) and a significant increase in interinfusion intervals (*P* < 0.05; Fig. 10G, right). In the presence of laser stimulation, mice self-administered less cocaine and needed more time to reach the maximum of 50 infusions of cocaine during daily 3-hour sessions.

To exclude the possibility that this reduction in cocaine self-administration was due to locomotor impairment after laser stimulation, we observed locomotor responses to cocaine in the presence or absence of laser stimulation. We found that stimulation of bilateral RNm glutamate terminals in the VTA altered neither basal open-field locomotor activity (fig. S10, A and B) nor cocaine-induced hyperlocomotion (10 mg/kg) (fig. S10C).

### Stimulation of VTA DA neurons inhibits cocaine self-administration

To further determine whether the above effects are mediated by activation of VTA DA neurons, we injected AAV-ChR2-eYFP into the VTA in DAT-Cre mice and then used the same laser stimulation to activate VTA DA neurons (Fig. 10H). We found that photoactivation of DA neurons produced a reduction in cocaine self-administration (Fig. 10I) in a manner similar to stimulation of RNm glutamate neurons (Fig. 10C) or of their projection terminals in the VTA (Fig. 10G). Paired *t* tests revealed a significant decrease in the total number of cocaine infusions (*P <* 0.05; Fig. 10I) and in the rate of cocaine infusions (*P <* 0.05) and an increase in interinfusion intervals (*P <* 0.05).

### Stimulation of VTA glutamate neurons failed to alter cocaine self-administration

We note that local glutamate neurons have been found within the VTA (*38*), which may project locally onto VTA DA neurons and functionally modulate DA neuronal activity (*39*). To determine whether the viruses used in the present experiments might diffuse from the RNm (the injection site) into the VTA where AAV-ChR2-eYFP might be expressed in VTA glutamate neurons, we directly injected AAV-ChR2-eYPF into the VTA in VgluT2-Cre mice. We found that photoactivation of VTA glutamate neurons failed to alter cocaine self-administration (fig. S11, A and B).

### Stimulation of GABA neurons in the RN failed to alter cocaine self-administration

We also examined the effects of optical manipulation of RNm GABAergic neurons on cocaine self-administration. To this end, the AAV vectors were injected bilaterally into the RNm to express ChR2 or halorhodopsin in GABA neurons in Vgat-Cre mice. No differences were observed when using either 473-nm blue laser stimulation to activate (fig. S11, C and D) or 532-nm green laser stimulation to inhibit RNm GABAergic neurons (fig. S11, E and F). This is consistent with our histological findings that very few GAD1^+^ GABAergic neurons are found in the RNm (Fig. 2E and fig. S3).

### Stimulation of glutamate neurons in the hippocampus failed to alter cocaine self-administration

As described earlier, wheel-running increased c-fos expression in the ventral hippocampus (vHipp) in addition to the RN (fig. S12A). To address whether the vHipp also contributes to the therapeutic anticocaine effects of exercise, we measured the effect of laser stimulation of vHipp glutamate neurons on cocaine self-administration (fig. S12, B and C). Bilateral vHipp stimulation failed to alter cocaine self-administration (fig. S12D).

## DISCUSSION

The major finding in this study is that physical exercise can activate a subset of glutamate neurons in the RNm that project to VTA DA neurons, producing rewarding effects as assessed by optical CPP and oICSS. We demonstrated that interruption of this RN-VTA glutamate pathway blocked wheel-running exercise reward. In addition, wheel-running exercise or direct stimulation of this RN-VTA pathway produced a significant decrease in cocaine intake during cocaine self-administration and a reduction in cocaine seeking during extinction. These findings suggest that activation of this pathway, at least in part, underlies the therapeutic benefit of physical exercise against cocaine abuse. Regarding the interactions between motor and reward function, previous studies have focused on whether drugs of abuse produce locomotor effects by activation of reward circuits, while little attention has been paid to how physical activity alters the mesolimbic DA system, producing exercise reward. In the present study, we identified and characterized a specific RN-VTA glutamate pathway that integrates the motor and reward systems. It appears that this is a bidirectional interaction. That is, physical activity may initially activate a subpopulation of glutamate neurons in the RN, which subsequently activates DA neurons in the VTA. These findings provide fresh insight into our understanding of how exercise produces rewarding effects, a conundrum that has existed in this field for decades.

### Wheel-running as a model for studying exercise reward

Voluntary wheel-running is rewarding in laboratory rodents and has served as a preclinical model of voluntary exercise in humans for decades (*40*). Rats and mice engage in wheel-running spontaneously and robustly, covering 5 to 6 km/day (when offered unlimited access to running wheels) over the course of 14 days (*22*), with females running on average 1.5 times more and faster than males (*3*, *40*). Running in wild animals is a means of exploring food, water, sexual partners, or escaping risk (*5*, *41*). Hence, being physically active is essential for survival (*42*) and is intrinsically motivating (*5*). In addition, recent studies indicate that rodents perform operant responses for access to a running wheel, prefer an environment associated with running (*7*), and demonstrate increased running behavior after a period of forced running wheel abstinence (*43*), suggesting that wheel-running is highly rewarding (*5*, *23*). However, the neural mechanisms underlying exercise reward and other beneficial effects of exercise have been poorly understood.

### Neural mechanisms underlying exercise reward

Previous studies using c-fos expression to identify brain regions involved in exercise (forced treadmill running) have implicated the lateral hypothalamus, cuneiform nucleus, pedunculopontine tegmental nucleus, pontine nucleus, lateral periaqueductal gray, caudate-putamen, and hippocampus (*44*, *45*). By using selectively bred high runner mice, it was found that this strain displayed a reduction in basal DA transmission (*46*), while wheel-running significantly enhanced c-fos response in such brain regions as the prefrontal cortex (PFC), entorhinal cortex, nucleus accumbens (NAc), dorsal striatum, and lateral hypothalamus, as compared to randomly bred control mice (*47*, *48*). Long-term voluntary exercise has been associated with increases in DA release and ΔFosB/FosB expression in the NAc and in TH mRNA levels in the VTA and substantia nigra pars compacta (SNc), as well as a reduction in DA D2 mRNA in the NAc core (*3*, *49*), suggesting that the mesolimbic DA system might be involved in exercise reward (*3*). However, until now, it was unknown how voluntary wheel-running activates the mesolimbic DA system.

In the present study, we employed a widely used wheel-running procedure (*50*) to map brain regions activated by wheel-running with a special focus on midbrain structures. We found that prolonged (10 days) daily voluntary wheel-running significantly increased c-fos expression in multiple brain regions, including the PFC, NAc, dorsal striatum, and hippocampus. However, what interested us most was the robust c-fos expression in the RNm, a critical motor nucleus adjacent to the VTA in the midbrain, suggesting the possible involvement of RNm in exercise reward. In line with this unexpected finding, we also found that optogenetic stimulation of RNm glutamate neurons is rewarding, as assessed by oICSS and laser-paired CPP. This finding suggests that the RNm is involved not only in motor function but also in reward and motivational behavior. This is supported by early findings that RN neuron activation and synaptic plasticity contribute to the acquisition of classically conditioned eyelid responses in rabbits (*30*, *51*). It is also supported by human functional magnetic resonance image studies showing that the RN is involved in a motivational motor task (*52*), in the pleasure of itch relief (*53*), and in anticipatory pleasure (*54*).

Another important finding in the present study is that a subgroup of RNm glutamate neurons project to the VTA and synapse onto DA neurons. It is well known that the rubrospinal tract, a descending motor pathway originating in the contralateral RN, travels through the VTA (*29*). Both the RN and VTA have been suggested to be functionally involved in regulating motor and reward functions, but independently—without interaction at the midbrain level. However, midbrain DA neurons and RN neurons share the same developmental origin (*55*). Here, we used cell type–specific neural imaging, tract-tracing, electrophysiology, and optogenetic approaches to address this question. We found that ~20% of glutamate neurons project from the RNm to VTA and functionally regulate DA neuronal activity and reward-driven behavior as assessed by oICSS and CPP. In addition, we found that DA neurons in the VTA project locally to the RN, suggesting that VTA DA neurons may functionally regulate RN neuronal activity and motor behavior. This is supported by the finding that D2 and D3 receptors and the DA transporters are detected in RN neurons within human brain tissue (*56*). Further functional assays indicate that DA produces an inhibitory effect on the excitability of RN neurons in the baboon brain (*57*). Together, these findings challenge the long-held view that the descending rubrospinal tract simply travels through the VTA to regulate motor neurons in the spinal cord, without functional connections to VTA neurons. In contrast to this view, we found that some, but not all, RN glutamate neurons directly project to VTA DA neurons and functionally regulate DA neuron activity and reward behavior. Congruent with the above findings, prolonged wheel-running also increased c-fos expression in VTA DA neurons. An early study failed to detect c-fos expression in VTA DA neurons after a shorter period of wheel-running (*3*), suggesting that c-fos expression in DA neurons is exercise intensity dependent. We should point out that in addition to physical exercise, prior Pavlovian conditioning with other nonrewarding stimuli may also activate the RN-VTA glutamate pathway, causing a reduction in later cocaine self-administration. As stated above, prolonged exercise also stimulates the release of β-endorphins and endocannabinoids that may subsequently modulate the mesolimbic DA system. These findings suggest that multiple neural mechanisms may underlie exercise-induced activation of the mesolimbic DA system, exercise reward, and therapeutic effects against drug abuse and addiction. The present findings indicate that the RN is critically involved in both exercise reward and drug reward.

We also note that bilateral optical stimulation of glutamate neurons in the RNm did not significantly alter basal open-field locomotion. This was an unexpected finding since the RN is a critical motor structure, and locomotor-stimulating effects would be predicted after activation. One possible explanation is that bilateral stimulation of the RNm may produce locomotor effects in opposite directions, leading to a mutual cancelation of opponent effects and no significant final net locomotor behavior. This is supported by our recent finding that unilateral, but not bilateral, stimulation of RNm glutamate neurons produces robust contralateral turning behavior in VgluT2-Cre mice that received intra-RN AAV-ChR2 microinjections (*58*).

### Neural mechanisms underlying the anti-addictive effects of exercise

Exercise is beneficial both physically and psychologically (*59*). Epidemiological studies have consistently shown an inverse relationship between exercise and substance use disorders (*36*). Extensive preclinical evidence indicates that various forms of physical exercise decrease self-administration of drugs of abuse (such as cocaine, nicotine, and opioids) (*8*, *60*, *61*). Therefore, active physical exercise has been recommended as a potentially effective intervention against drug abuse and addiction (*4*, *62*).

Several hypotheses have been proposed to explain how physical exercise is efficacious in the adjunctive treatment and prevention of substance abuse. A commonly held view is that physical exercise and drugs of abuse may act on a common neural circuit—the mesolimbic DA system—and therefore alter behavioral responses to drugs of abuse. For example, exercise may normalize hypofunctionality in the DA system in drug users by altering neurotransmitter release and the level of transcription factors in the NAc (*35*, *63*), thereby decreasing DA neuronal response to drugs of abuse. This is supported by previous research showing that exercise may prevent the initiation of drug use and abuse by interactions with DA, and prevent relapse by interactions with glutamate and DA in the reward circuitry of the brain (*35*). However, no prior work has addressed how the reward circuit is activated in response to physical exercise and integration of the mesolimbic and motor systems during exercise.

In the present study, we found that wheel-running robustly activates RNm glutamate neurons that project to the VTA and functionally potentiate DA neuronal activity, while inhibition of the RN-VTA glutamate pathway attenuates wheel-running behavior. In a cocaine versus wheel-running choice test, animals displayed both cocaine self-administration and wheel-running. However, mice with higher levels of wheel-running took substantially less cocaine. There was a significant negative correlation between wheel-running activity and cocaine self-administration, suggesting that wheel-running inhibits cocaine self-administration. Notably, direct stimulation of the RN-VTA glutamate pathway (to mimic wheel-running action) inhibits cocaine self-administration in a manner similar to wheel-running, suggesting that wheel-running likely reduces cocaine intake by activation of the RN-VTA glutamate pathway that subsequently activates the mesolimbic DA system. These findings plausibly explain—at both the neuro-circuitry and cellular levels—how physical exercise produces its protective effects against drug use and abuse.

The precise mechanisms through which exercise inhibits cocaine self-administration are still unclear. However, on the basis of the extinction-like pattern of cocaine self-administration with running wheel access (Fig. 8), it appears that exercise produces a reduction in cocaine’s rewarding effects. Animals given intensive physical exercise in the wheel-running paradigm seem to display less interest in cocaine—possibly due to exercise-induced partial activation of the mesolimbic DA system, resulting in decreased DA response to cocaine. This is consistent with finding that exercised animals display a decreased locomotor response to novelty or morphine (*64*).

Unexpectedly, we found that wheel-running exercise produced a long-lasting increase in glutamate transmission from the RNm to VTA DA neurons quantified by an increased AMPAR/NMDAR ratio. Consistent with this finding, laser stimulation of RN-VTA glutamate terminals in the VTA produced a long-lasting CPP (up to 3 days). Similarly, wheel-running during acquisition of cocaine self-administration induced a long-lasting reduction in cocaine seeking under extinction conditions in the absence of the running wheel or when the wheel was locked. The mechanisms underlying these persistent effects are unclear. However, it is likely that Pavlovian conditioning is involved. For instance, the act of entering and exiting the locked wheel might have produced a conditioned response and suppressed drug-seeking behavior. Regardless, enhanced glutamate transmission from the RN to the VTA likely mediates the long-lasting effects. In other words, the RN-VTA glutamate pathway may be highly sensitive to physical exercise or direct laser stimulation.

This effect is similar to the increases in synaptic glutamate transmission in the VTA observed following cocaine administration, which also last for several days after a single injection (*65*). Overall, the present study suggests that regular and intensive physical activity is protective against cocaine use by activation of the RN-VTA glutamate pathway. Thus, this pathway may constitute a new therapeutic target for the prevention and treatment of substance use disorders. Given that the mesolimbic DA system is involved in other central nervous system disorders and exercise can alleviate symptoms of depression, anxiety, and obsessive-compulsive disorder, our findings suggest that the same RN-VTA glutamate mechanism may underlie the beneficial effects of exercise in a broader sense.

As a final point of interest, we observed significant sex differences in wheel-running, c-fos response to exercise, cocaine self-administration, and the effects of exercise on cocaine self-administration. Females ran more than males when the running wheels were freely available (unlocked) during the light cycle, as reported previously (*4*). Female mice also displayed much higher c-fos responding in RN glutamate neurons after wheel-running and higher reduction in cocaine self-administration than males. These findings suggest that physical exercise decreases vulnerability to drugs of abuse and that females display higher sensitivity to its reward-substitution effects. Thus, gender is an important factor to be considered in the prevention and treatment of substance use disorders (*4*).

We should point out that the present findings are a rudimentary assessment of how exercise produces rewarding and potentially therapeutic and beneficial effects and many questions remain to be addressed. For example, we do not know whether the glutamate projections from the RN to VTA DA neurons are direct or via collaterals of the rubrospinal tract passing through the VTA; whether RN glutamate neurons also synapse onto DA neurons in the SNc, another critical brain region involved in both motor and reward functions; and whether RN glutamate neurons synapse onto other non-DA neurons in the VTA or SNc, or project to other brain regions such as the NAc. It is also unclear whether GABAergic neurons in the RNp also project onto midbrain DA or non-DA neurons and why the patterns of cocaine self-administration after wheel-running exercise versus optical stimulation are different. More studies are required to resolve these questions and increase our understanding of the RN’s role in locomotion and brain reward.

In conclusion, the present study used a prolonged daily voluntary wheel-running model to explore the neural mechanisms underlying exercise reward and its anticocaine effects. We found that wheel-running significantly activated RNm glutamate neurons that project to the VTA, particularly to contralateral VTA DA neurons. Activation of this pathway produced rewarding effects, while inhibition of this pathway attenuated wheel-running reward, suggesting that the RNm-to-VTA glutamate pathway, at least in part, underlies exercise reward. In addition, wheel-running or direct stimulation of this pathway inhibited cocaine self-administration and decreased cocaine intake, suggesting that this newly identified circuit may partially mediate the beneficial effects against cocaine use disorder. Given that wheel-running modulates not only DA and glutamate levels but also endogenous opioids, endocannabinoids, ghrelin, and leptin that in turn modulate the mesolimbic DA system, the present findings expand our understanding of how exercise produces such a wide range of therapeutic benefits, including antiaddiction, antidepressive, antianxiety, and antiobesity effects.

## MATERIALS AND METHODS

### Animals

Male and female VgluT2-IRES-Cre^+/−^ (VgluT2-Cre), DAT-IRES-Cre^+/−^ (DAT-Cre), and Vgat-IRES-Cre^+/−^ (Vgat-Cre) mice and wild-type (WT) mice with C57B/6 genetic background, aged 8 to 16 weeks, were bred at the National Institute on Drug Abuse (NIDA), Intramural Research Program and used in the present study. WT mice (Charles River Laboratories, Raleigh, NC, USA) were used for ISH, c-fos, and FG/RB retrograde tracing histological experiments. The heterogeneous offspring of three Cre-driven lines obtained from The Jackson Laboratory (Bar Harbor, ME, USA) [DAT-Cre (B6.SJL-*Slc6a3*^tm1.1(Cre)Bkmn^/J), stock #020080; Vgat-Cre (B6J.129S6(FVB)-*Slc32a1*^tm2(cre)Lowl^/MwarJ), stock #028862; VgluT2-Cre (*Slc17a6*^tm2(cre)Lowl^/J, stock #016963] were used for electrophysiological and neuron type–specific behavioral experiments. The Cre transgenic lines were bred for >10 generations on the background of C57BL/6 mice from Charles River Laboratories (Frederick, MD, USA). All animals were housed individually in single cages after surgery and allowed to acclimatize to a reverse 12/12-hour light-dark cycle condition (lights off at 8:00 a.m.) with food and water freely available in their home cages. The experimental procedures followed the *Guide for the Care and Use of Laboratory Animals, 8th edition* (National Research Council of the U.S. National Academy of Sciences) and were approved by the Animal Care and Use Committee (ACUC) of the NIDA and the ACUC of Medical College of Wisconsin.

### Wheel-running

Wheel-running exercise was performed using mouse wireless running wheels purchased from Med Associates (ENV-047, St. Albans, VT, USA), which can fit in mouse home cages and mouse drug self-administration chambers. Wheel-running activity data were recorded and analyzed with Wheel Manager software (Med Associates, SOF-860, St. Albans, VT, USA) and presented as the number of revolutions within a designated period of time. In the home cage training phase, WT mice that were assigned to the wheel-running group were allowed free access to the running wheel device in their home cages overnight (during the light cycle, ~12 hours) for 5 days for acclimation and acquisition, followed by an additional 5 days with daily 2-hour running during the dark cycle (for c-fos examination and electrophysiological studies), while the wheel-running procedures for the sedentary control mice were the same except the wheels were locked across all phases of the experiment (Fig. 1A).

To observe the effects of wheel-running on cocaine self-administration and determine whether the introduction of the wheel-running device might act as a novel object to alter cocaine self-administration, we used a wheel “unlocked-locked-unlocked-removed” sequential procedure during the cocaine self-administration and wheel-running choice sessions, with each phase lasting for three consecutive days (Fig. 1). In the “unlocked” phase, animals could choose wheel-running or lever pressing to receive a cocaine infusion, while in the locked phase the running wheels in the self-administration chambers were locked, and animals could stay on the wheels but not run. During the last 3 days, the running wheels were removed from the self-administration chambers. In addition, a “no wheel-wheel-unlocked-locked” procedure was used to study sexual difference in the cocaine-wheel-running choice test (Fig. 9). Animals were then decapitated and their brains were collected immediately (within 1 hour) after the last session of wheel-running for c-fos immunostaining determination.

### Stereotaxic surgery

Intracranial stereotaxic surgery was performed for the injections of viral vectors, anterograde or retrograde neuroanatomical tracers, and optrode implantation. Briefly, animals were anesthetized by intraperitoneal injection of a ketamine/xylazine mixture (100/10 mg/kg) and then positioned in a stereotaxic frame (David Kopf Instruments, Tujunga, CA, USA) to fix the head with ear bars and an incisor adaptor. After exposing the top area of the animal skull, craniotomies were drilled at stereotaxic coordinates determined from a standard mouse brain atlas. Iontophoretic injection technique was used for retrograde tracing with FG or RB (red fluorescent RetroBeads, Lumafluor Inc., Durham, NC, USA) in WT mice. Specifically, the animals received an iontophoretic injection of 1% FG in cacodylate buffer (pH 7.4) unilaterally into the VTA [anterior-posterior (AP): −3.28 mm; medial-lateral (ML): ±1.2 mm; dorsal-ventral (DV): −4.47 mm] or the RNm (AP: −3.64 mm; ML: ±1.14 mm; DV: −3.81 mm, angle 10° from midline) at a 1-μA current with 7-s pulses for 15 min via a glass capillary tube.

All viral vectors used in the present study were obtained from the Vector Core at the University of North Carolina. To perform the intracranial microinjection of Cre-inducible AAV for behavioral and electrophysiological experiments, the microinjection pump and a 10-μl NanoFil syringe with a 33-gauge beveled needle were used for delivering the purified viral vectors. AAV5-EF1α-DIO-hChR2-eYFP (AAV-ChR2-eYFP) and AAV5-EF1α-DIO-eNpHR3.0-eYFP (AAV-NpHR-eYFP), which encode ChR2 or eNpHR3.0 (halorhodopsin) in the presence of Cre recombinase, were used respectively to optically activate or inhibit neuronal activity of specific neurons in brain regions of interest. To specifically target VgluT2^+^ neurons in the RNm, 0.2 μl of AAV-ChR2-eYFP or AAV-NpHR-eYFP was bilaterally infused into the RN (AP: −3.64 mm, ML: ±1.14 mm, DV: −3.81 mm, angle 10° from midline) in VgluT2-Cre mice. For cell type–specific targeting in the VTA, DAT-Cre, VgluT2-Cre, and Vgat-Cre mice received 0.15 μl of virus bilaterally into the VTA (AP: −3.28 mm; ML: ±1.2 mm; DV: −4.47 mm, angle 10° from midline). The virus was injected over 5 min, and the needle was left in place for an additional 5 min to prevent backflow of virus up the needle track. The animal was then bilaterally implanted with optical fibers into the RNm or VTA with ceramic ferrules (0.2 mm above the injection site), which were connected to a split patch cord (Doric Lenses Inc., L’Ancienne-Lorette, QC, Canada) by ceramic sleeves for receiving laser stimulation, and secured with dental cement to the top of the skull.

### RNAscope ISH

RNAscope multiplex fluorescent assays (Advanced Cell Diagnostics, Newark, CA, USA) were performed to examine multiple gene transcript expression in the RN. Whole brains were rapidly harvested and frozen on dry ice from deeply anesthetized C57BL/6 mice. Then, the brain tissue was sliced into 12-μm-thick sections at −18°C in a cryostat (Leica CM3050S) followed by immediate mounting onto Fisher Scientific SuperFrost Plus Slides. According to the manufacturer’s manual, the brain slices were sequentially prepared by fixation in 10% neutral buffered formalin, ethanol dehydration, and then protease pretreatment. The following probes (purchased from Advanced Cell Diagnostics, Newark, CA, USA) were used to detect *FOS* (c-fos), *Scl17a6* (VgluT2), *GAD1*, and *TH* mRNA expression and their colocalization following the instructions: the FOS RNAscope probe [catalog no. 316921, targeting *Mus musculus* FBJ osteosarcoma oncogene (*FOS*) mRNA, NM_010234.2], the *Slc17a6* RNAscope probe [catalog no. 319171-C3, targeting 1986 to 2998 base pairs (bp) of the *M. musculus Slc17a6* mRNA sequence, NM_080853.3], the *TH*-specific RNAscope probe (catalog no. 317621-C2, targeting 483 to 1603 bp of the *M. musculus TH* mRNA sequence, NM_009377.1), and the *GAD1*-specific RNAscope probe (catalog no. 400951-C3, targeting 62 to 3113 bp of NM_008077.4). Fluorescence of the target RNAs was visualized, and images were taken using a confocal microscope (Olympus Fluor View FV1000, Olympus Corporation, Tokyo, Japan). The c-fos^+^ (and RB^+^ and VgluT2^+^) neurons in the RN and VTA were counted in three to seven stereologically selected sections per brain with 100-μm spacing between sections from the rostral to the caudal level under ×20 magnification.

### Immunohistochemistry

To visualize the expression of virus in the brain, mice were fully anesthetized using a ketamine/xylazine mixture in 0.9% (w/v) saline solution (intraperitoneally) before transcardial perfusion with 0.9% saline, followed by 4% (w/v) paraformaldehyde (PFA) in 0.1 M phosphate buffer (PB; pH7.4). The brains were rapidly removed from the skull, postfixed in 4% PFA at 4°C with shaking for an additional 2 hours, and transferred to 20% (w/v) and then to 30% (w/v) sucrose/PB solution to allow serial dehydration. After that, the brains were frozen in a −80°C freezer for storage or further processing on a cryostat. The brain samples were sectioned coronally at a thickness of 20 μm and collected in PB. Free-floating sections were incubated in blocking buffer (4% bovine serum albumin, in PB supplemented with 0.3% Triton X-100) at room temperature for 1 hour before incubation with the primary antibodies in blocking buffer. A mouse anti-TH antibody (1:500; Millipore AB318) was added to detect the DA neuronal marker TH. A mouse anti–c-fos antibody (1:500; Abcam, ab208942) and a rabbit anti-TH antibody (1:500; Millipore, AB152) were used in the immunohistochemistry assay to measure c-fos expression in the midbrain. To increase the FG signals, anti-FG antibody (1:5000; Millipore, AB153-I) was used to label the FG^+^ cells. After incubating with primary antibody overnight, the sections were rinsed three times for 10 min in PB under gentle agitation and incubated for 2 hours at room temperature with Alexa Fluor–conjugated secondary antibodies from Life Technologies (Thermo Fisher Scientific, Waltham, MA, USA) at 1:500 dilution. Last, the slices underwent three 10-min rinses before being mounted onto glass slides and coverslipped with Fluoro-Gel mounting medium. Imaging was processed using an Olympus Fluor View FV1000 confocal microscope.

### Electrophysiology recording

Mice were anesthetized with isoflurane, and the brain was removed rapidly from the skull and placed in an *N*-methyl-d-glucamine (NMDG) slicing solution (containing 92 mM NMDG, 2.5 mM KCl, 30 mM NaHCO_3_, 1.2 mM NaH_2_PO_4_, 20 mM Hepes, 25 mM glucose, 5 mM sodium ascorbate, 2 mM thiourea, and 3 mM sodium pyruvate, adjusted to pH 7.4 with HCl) and sliced using a vibratome (VT1200S, Leica, Wetzlar, Germany). Horizontal midbrain slices (150 to 250 μm) containing RNm or VTA were allowed to stabilize for 10 min at 33°C in NMDG solution, and then at least 30 min at room temperature in a modified Hepes artificial cerebrospinal fluid (aCSF; containing 92 mM NaCl, 2.5 mM KCl, 30 mM NaHCO_3_, 1.2 mM NaH2PO_4_, 20 mM Hepes, 25 mM glucose, 5 mM sodium ascorbate, 2 mM thiourea, and 3 mM sodium pyruvate) before recordings. Slices were transferred to the recording chamber and perfused with standard aCSF (containing 125 mM NaCl, 2.5 mM KCl, 2 mM CaCl_2_, 1 mM MgCl_2_, 26 mM NaHCO_3_, 1.25 mM NaH_2_PO_4_, and 11 mM glucose) bubbled with 95% O_2_/5% CO_2_ for whole-cell patch-clamp recordings, performed at 30° to 32°C. Signals were amplified with a Multiclamp 700B amplifier, acquired using a Digidata 1440A digitizer, sampled at 10 kHz, and filtered at 2 kHz. All data acquisition and analysis were performed using pCLAMP software (Molecular Devices, Sunnyvale, CA, USA). Neurons were visually identified using infrared differential interference contrast and fluorescence microscopy. In some experiments, biocytin was included within the patch electrode for marking and filling cells during the recording session. Where these injections were successful, recorded neurons were morphologically characterized. Series resistance (15 to 25 megohms) and/or input resistance were monitored online with a 5-mV hyperpolarizing step (50 ms) given between stimulation sweeps. The resistance of the patch pipettes was 2.0 to 3.5 megohms when filled with intracellular solution.

To record action potentials, we used an intracellular solution containing the following: 135 mM K-gluconate, 4 mM KCl, 10 mM Hepes, 4 mM MgATP, 0.3 mM Na_2_GTP, and 10 mM Na_2_-phosphocreatine (pH was adjusted to 7.2 with KOH). For current-clamp experiments to characterize cell firing, 10 pulses at frequencies of 1, 5, 10, 20, and 40 Hz were tested to determine spike fidelity (the percentage of light pulses that lead to action potentials). For all voltage-clamp experiments, we used an intracellular solution containing the following: 130 mM Cs-methanesulfonate, 10 mM CsCl, 10 mM Hepes, 1.1 mM EGTA, 2 mM MgCl_2_, 4 mM MgATP, 0.3 mM Na_2_GTP, and 10 mM Na_2_-phosphocreatine (pH 7.2 with CsOH). RNm ChR2-labeled glutamatergic neurons or VTA DA neurons were voltage-clamped at −70 mV. Optical stimulation was used (pulses of 5 mW, 473-nm light delivery via a 62.5-μm core fiber, submerged in the bath and aimed at the region of interest) to evoke presynaptic glutamate release from RN projections to the VTA. The GABA_A_ blocker picrotoxin (50 μM) was added to the extracellular solution for pharmacological characterization of glutamate currents. Optogenetically evoked EPSCs (oEPSCs) were recorded for 10 min followed by bath application of 10 μM CNQX and 50 μM AP5 for an additional 10 min. Ten to 12 sweeps before and after drug were averaged, and peak oEPSC amplitudes were then measured. For measuring AMPAR/NMDAR ratio, VTA DA neurons were voltage-clamped at +40 mV to record total oEPSCs containing both AMPAR and NMDAR components. After a stable baseline recording of total oEPSCs, the NMDAR antagonist (*R*)-CPP (10 μM) was applied in the bath for 6 to 10 min to isolate fast AMPAR-oEPSCs. NMDAR-oEPSCs were calculated by subtracting AMPAR-oEPSCs from the total oEPSCs in the same neuron. An average of 10 to 20 consecutive oEPSCs was collected for each type of EPSC. AMPAR/NMDAR ratio was calculated by dividing the peak of the AMPAR-oEPSCs by the peak of the NMDAR-oEPSCs.

### Optical intracranial self-stimulation

To study whether activation of the RNm-VTA glutamatergic pathway is rewarding, optogenetic techniques were used to specifically activate cell bodies of RNm glutamate neurons, their projection terminals in the VTA, or their downstream VTA DA neurons using oICSS. Three groups of Cre transgenic mice were used: (i) VgluT2-Cre mice expressing ChR2 in RNm glutamatergic neurons with optic fibers implanted in the same area for directly stimulating RNm glutamate cell bodies, (ii) VgluT2-Cre mice with intra-RNm AAV-ChR2-eYFP microinjections and intra-VTA optic fiber implantation for optical stimulation of RNm glutamate terminals, and (iii) DAT-Cre mice with both intra-VTA AAV-ChR2-eYFP microinjections and optic fiber implantation for photoactivation of VTA DA neurons.

oICSS training was conducted in mouse operant response chambers (Med Associates Inc., St. Albans, VT, USA) equipped with two retractable levers (active and inactive), a house light, and cue lights above each lever. Eight weeks after stereotaxic surgery, the animals were placed in the chambers for daily behavioral training. A laser generator (OEM Laser System, Midvale, UT, USA) was triggered by a Master-9 pulse stimulator (A.M.P.I., Jerusalem, Israel). During each 60-min oICSS training session, a train of laser pulses (473 nm, 10-ms duration per pulses, 20 Hz, 40 pulses, 10 mW) was delivered upon each active lever press by the mouse and the cue light was activated; inactive lever responses had no consequence. The numbers of active lever presses and inactive lever presses were recorded in each session. All mice were trained for at least 20 daily consecutive sessions, and then an active/inactive lever switch took place; training was conducted for the following 10 days. Scheduling of experimental events and data collection was accomplished using the Med-Associates Inc. software.

### Optical CPP

To rule out the possibility that the light of the laser and the cue light visually distracted the animals from oICSS behavior and produced an experimental confound, unbiased CPP was used to assess the rewarding effect of laser stimulation of the RN-to-VTA pathway. Experiments were conducted in the same three groups as the oICSS experiments. A three-compartment mouse CPP apparatus (Med-Associates Inc., St. Albans, VT, USA) with different wall colors and floor texture in each of the compartments and an Any-Maze video-tracking system (Stoelting Co., Wood Dale, IL, USA) was used to record the time each animal spent in each of the compartments during testing sessions.

The CPP procedure was conducted in the following three phases.

#### [Day 1] Preconditioned phase

Individual mice were placed in the center corridor and allowed free access to the entire CPP apparatus for 15 min, and baseline preferences were assessed by recording the time spent in each compartment.

#### [Days 2 to 4] Light-conditioned phase

In the morning, the animals were confined in the white-wall chamber with rod grid floor or black-wall chamber with meshed grid floor (counterbalanced) for 15 min and received 5-min laser pulses (473 nm, 10-ms duration per pulse, 20 Hz, 1 mW); 6 hours later during the afternoon of the same day, the mice were placed into the opposite black-wall chamber with meshed grid floor or white-wall chamber with rod grid floor (non–cue light–paired compartment) without laser stimulation.

#### [Day 5 or days 5 to 15] Posttest phase

Similar to day 1, the mice were placed in the center compartment and allowed free access again to the other two compartments, with video recording of time spent in each of the compartments. Chamber preference was assessed by the CPP score, which was the time (in seconds) spent in the laser-paired compartment minus time spent in the laser-unpaired compartment.

### Intravenous cocaine self-administration

Three groups of mice were used for intravenous cocaine self-administration experiments. Before the intravenous surgery, the mice underwent an intracranial AAV microinjection to express ChR2 or halorhodopsin in either RNm glutamate neurons in VgluT2-Cre mice, DA neurons in DAT-Cre mice, or GABAergic neurons in Vgat-Cre mice. Three weeks after AAV injection, the animals received jugular vein catheterization surgery for intravenous cocaine self-administration and intracranial optic fiber implantation surgery for optical stimulation. The surgical procedures for AAV injection, catheterization, and optrode implantation were the same as described previously (*32*). Briefly, mice were anesthetized with ketamine/xylazine mixture (100 mg/10 mg/kg, intraperitoneally). Incisions were made on the head to expose the calvarium and in the neck to expose the right external jugular vein. Catheterization was performed by tunneling subcutaneously from top incision to neck incision and inserting a microrenathane catheter (Braintree Scientific Inc., Braintree, MA, USA) into the jugular vein at a length of 1.2 cm. The incision in the neck area was sutured, and the distal end of the catheter was connected to a bent metal cannula (Plastics One, Roanoke, VA, USA), which was later secured to the skull together with the optical fibers by dental cement. The patency of the intravenous catheter was maintained by daily flushing using heparinized saline [30 United States Pharmacopera (USP) units/ml].

One week after the intravenous and intracranial surgeries, cocaine availability commenced in mouse self-administration chambers (Med-Associates Inc., St. Albans, VT, USA). The mice were individually placed into an operant chamber, their skull-mounted cannulae were connected to a syringe pump through a rotating liquid swivel (Med-Associates Inc., St. Albans, VT, USA) using tubing, and animals were allowed to freely explore inside their chambers. Under a fixed ratio 1 (FR1) schedule of reinforcement, the syringe pump was triggered by one active lever press, enabling the mice to receive 0.015 ml of cocaine solution (1 mg/kg per infusion) over 4.2 s each time, paired with a light and tone cue. Additional responses on the active lever during the infusion period or depression of the inactive lever were recorded but not reinforced. The animals were run in 3-hour sessions each day and were allowed a maximum of 50 infusions per daily session to prevent cocaine overdose when the cocaine dose was 1.0 mg/kg/infusion. After 1 to 2 weeks of cocaine self-administration, the cocaine dose was decreased to 0.5 mg/kg per infusion with a maximum of 100 infusions, leading to an increased number of active lever responses. High basal levels of responding allow us to see subtle experimental treatment–induced changes in drug-seeking behavior and therefore increase the sensitivity to an experimental treatment. After stable cocaine self-administration was achieved (defined as a minimum number of 20 cocaine infusions for three consecutive days, less than 10% variation in the number of total infusions, and a ratio of active versus inactive lever responses larger than 2:1), the laser stimulation was superimposed on the cocaine self-administration by connecting the animal’s optical fibers to a split patch cord, which was coupled to a hybrid optical/liquid rotary joint (Doric Lenses Inc., Ancienne-Lorette, QC, Canada) that allows an optical connection to the laser system and a liquid delivery from the syringe pump. The animals were allowed to habituate to the tethering for several days before testing until self-administration behavior restabilized. On the test day, animals were given photostimulation (473 nm, 10-ms duration per pulse, 20 Hz, 10 mW, 5 min on followed by 10 min off or 532 nm, 10 mW, 30 min on followed by 30 min off) throughout the 3-hour cocaine self-administration. The laser power intensity was minimized to prevent nonspecific locomotor impairment during laser stimulation.

### Locomotor activity

To observe the effect of laser stimulation on locomotor behavior, open-field locomotion was measured using an infrared activity monitor with removable Plexiglas chambers (Accuscan, Columbus, OH, USA). Mice were connected to the split patch cords and placed individually in separate test chambers to habituate to the apparatus for 1 hour/day for three consecutive days. On the following test day, the computer was programmed to record 30-min baseline followed by 30-min test behavior, with data collected at 5-min intervals. The laser pulses were delivered during the testing phase, with the same illumination parameter used during self-administration.

### Statistical analysis

Data analyses and graphing were accomplished using SigmaPlot software (version 12.5, Systat Software Inc.) and GraphPad Prism software (GraphPad Software Inc.). Animal group sizes were chosen on the basis of extensive previous experience with the animal models used. No data points were excluded from the analysis in any experiment. Where variation in group size occurred, this was due to animals being dropped from the experiment due to obstruction or clogging of intravenous catheters. The group size (*n* > 5) is the number of independent values (individual animals), which was sufficient to permit meaningful statistical analysis. Cell count comparisons between two groups were performed using two-tailed unpaired *t* tests. Paired *t* tests were used to compare between before and after measurements for the same group in laser-induced behavioral changes (CPP and cocaine self-administration) and the electrical activity of living neurons. One-way or two-way RM ANOVAs were used to evaluate wheel-running or laser stimulation–induced changes in open-field locomotion. Results were expressed as means ± SEM. *P* < 0.05 was considered a statistically significant difference.

## Notes

View/request a protocol for this paper from *Bio-protocol*.
