# Supplementary material for: A red nucleus–VTA glutamate pathway underlies exercise reward and the therapeutic effect of exercise on cocaine use
Source: Sci Adv. 2022 Sep 2;8(35):eabo1440. doi: 10.1126/sciadv.abo1440 (PMC10848951; doi:10.1126/sciadv.abo1440)
Supplement: Supplementary file 1 — Figs. S1 to S12 [file sciadv.abo1440_sm.pdf]

Science Advances
AAAS

Supplementary Materials for

A red nucleus–VTA glutamate pathway underlies exercise reward and the therapeutic effect of exercise on cocaine use

Yi He *et al.*

Corresponding author: Zheng-Xiong Xi, zxi@mail.nih.gov

Sci. Adv. **8**, eabo1440 (2022)
DOI: 10.1126/sciadv.abo1440

This PDF file includes:

Figs. S1 to S12

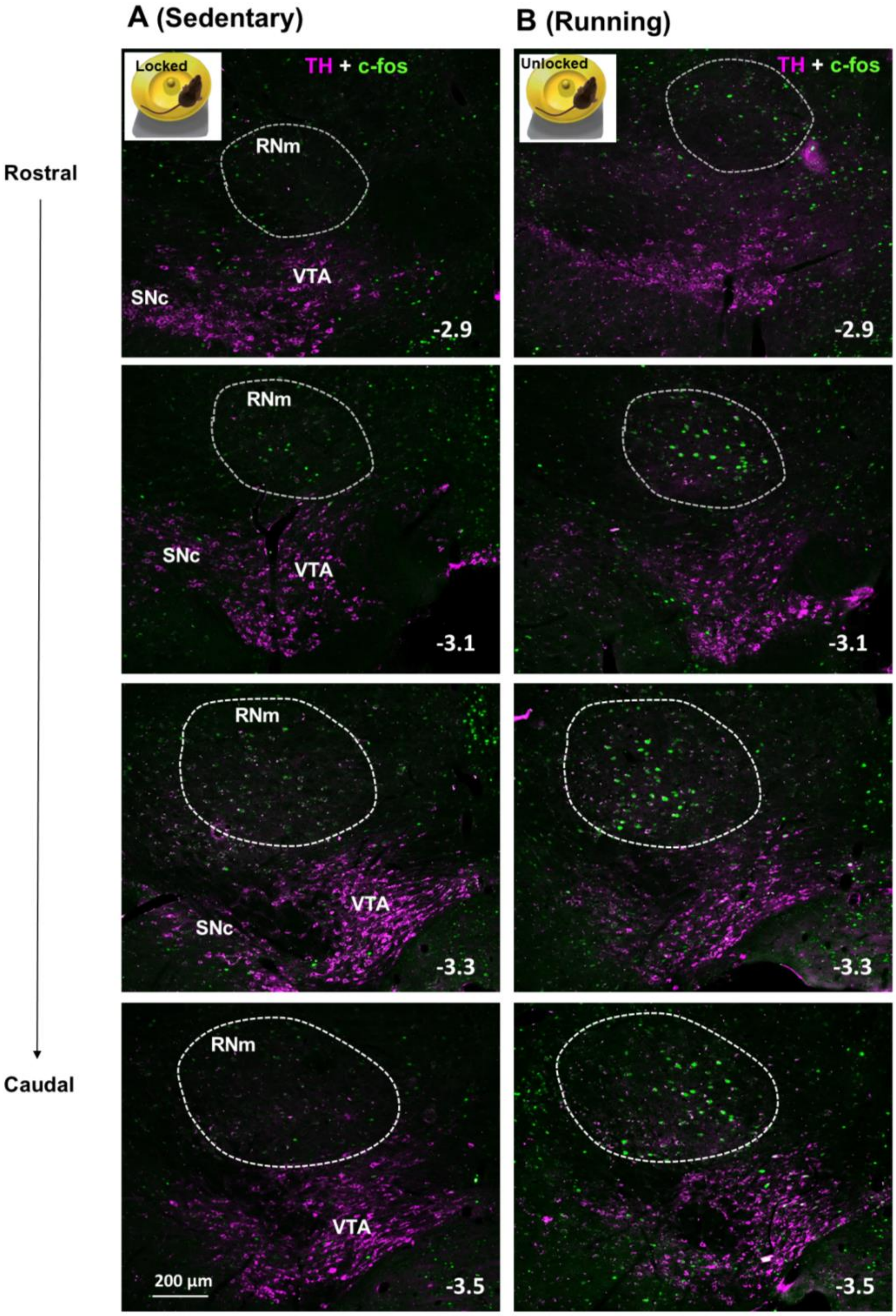

Figure S1 (related to Fig. 1): A series of images from the rostral to caudal levels of the RN/VTA. Wheel-running increased c-fos expression in the RNm relative to sedentary (wheel-locked) mice.

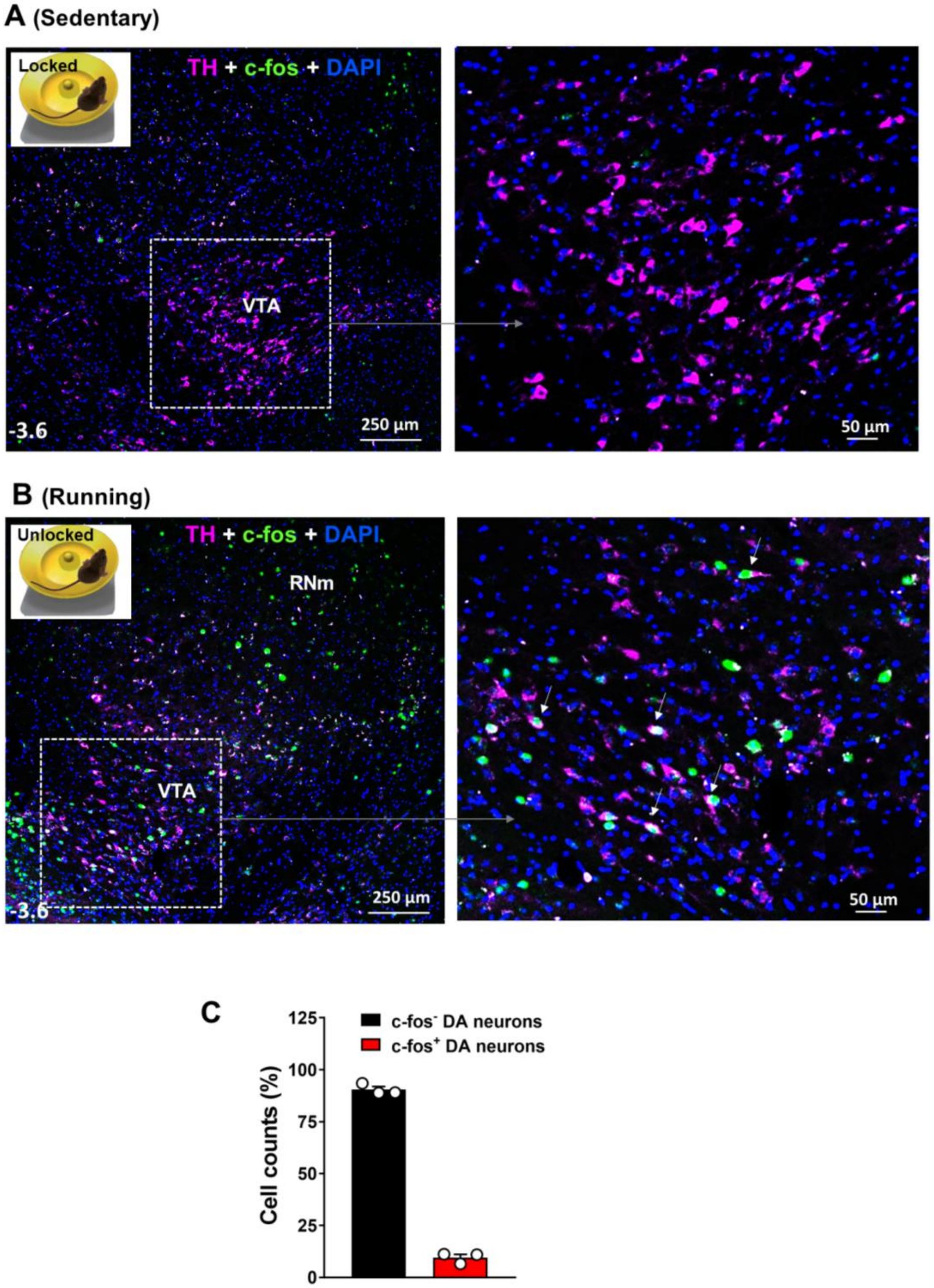

Figure S2 (related to Fig. 1): Wheel running increases c-fos expression in a subset of VTA DA neurons. ***A***: VTA c-fos expression in a sedentary control mouse when the wheel was locked; ***B***: VTA c-fos expression in a wheel-running mouse; ***C***: Quantitative cell counting indicates that ~10% of VTA DA neurons (marked by arrows) displayed c-fos expression after wheel-running.

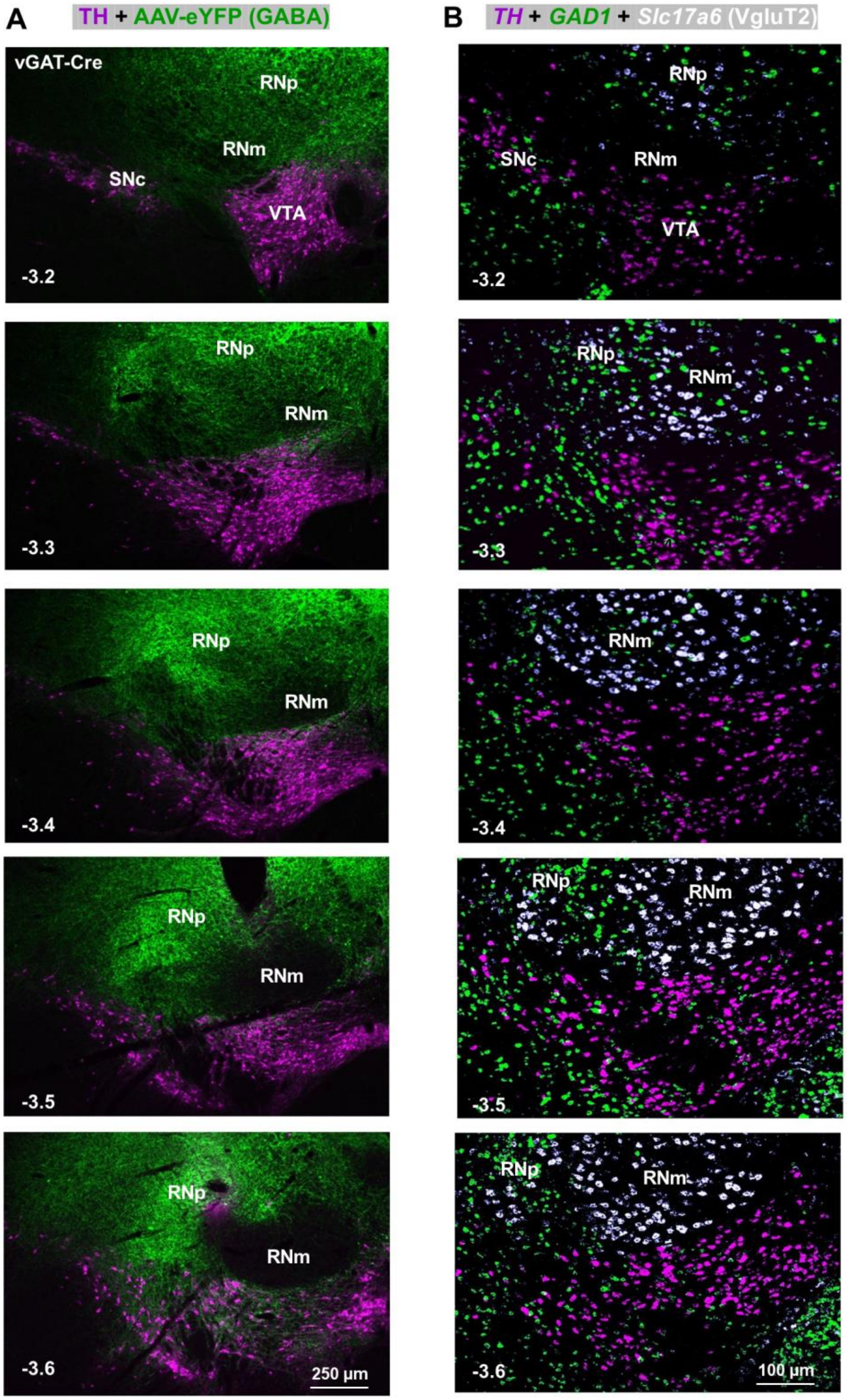

Figure S3 (related to Fig. 2): Series of brain slice images, illustrating GABAergic *vs.* glutamatergic neuronal distributions from the rostral RNp to the caudal RNm. *A*: AAV-

eYFP (green) and tyrosine hydroxylase (TH)-immunostaining. The AAV-eYFP vector was injected into the RN at Bregma -3.52 level in vGAT-cre mice to express eYFP in RN GABA neurons. GABAergic eYFP staining decreased progressively from the rostral RNp (Bregma -3.16) toward the caudal RNm (Bregma -3.64); ***B:*** Triple-staining RNAscope ISH assays, illustrating that *Slc17a6* (VgluT2)-positive (white) glutamate neurons progressively increased, while *GAD1*-positive GABA neurons (green) progressively decreased from the rostral RNp toward the caudal RNm. Midbrain DA neurons are labeled by TH-immunostaining (***A***) or *TH* mRNA (***B***) (red).

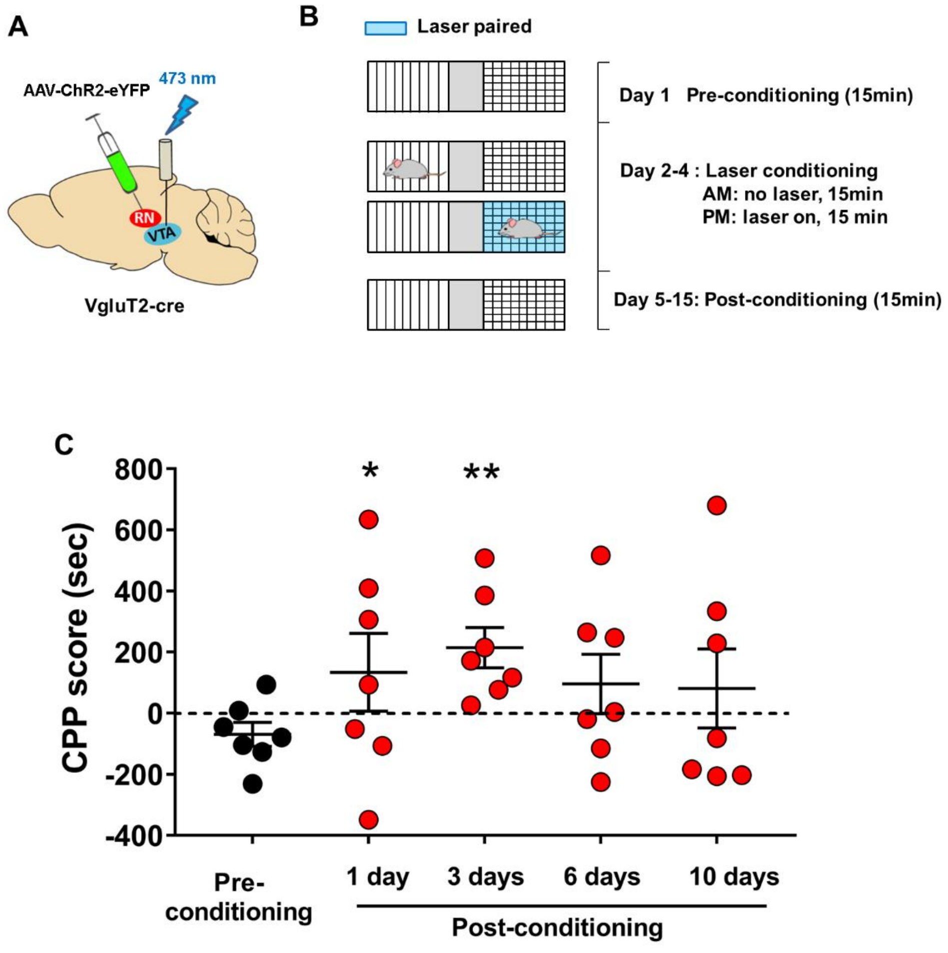

Figure S4 (related to Fig. 3): Optical conditioned place preference (CPP) produced by laser stimulation of RN-VTA glutamate projection terminals in the VTA. ***A***: A schematic diagram showing the general experimental methods. AAV-ChR2-eYPF was microinjected into bilateral RNm, while optical fibers were implanted to the VTA to stimulate ChR2-expressing glutamate terminals. ***B***: The sequence of 15 days of CPP procedures; ***C***: Three days of laser stimulation of RNm glutamate terminals in the VTA produced CPP in an additional group of mice, which lasted for 3 days. A one-way RM ANOVA analysis reveals significant laser treatment main effect ($F_{4,24}$=3.85, p<0.05). A post-hoc Dunnett's test indicated that CPP scores on day 1 and day 3 were significantly higher than those on the pre-conditioning session. *p<0.05, **p<0.01, as compared to pre-conditioning.

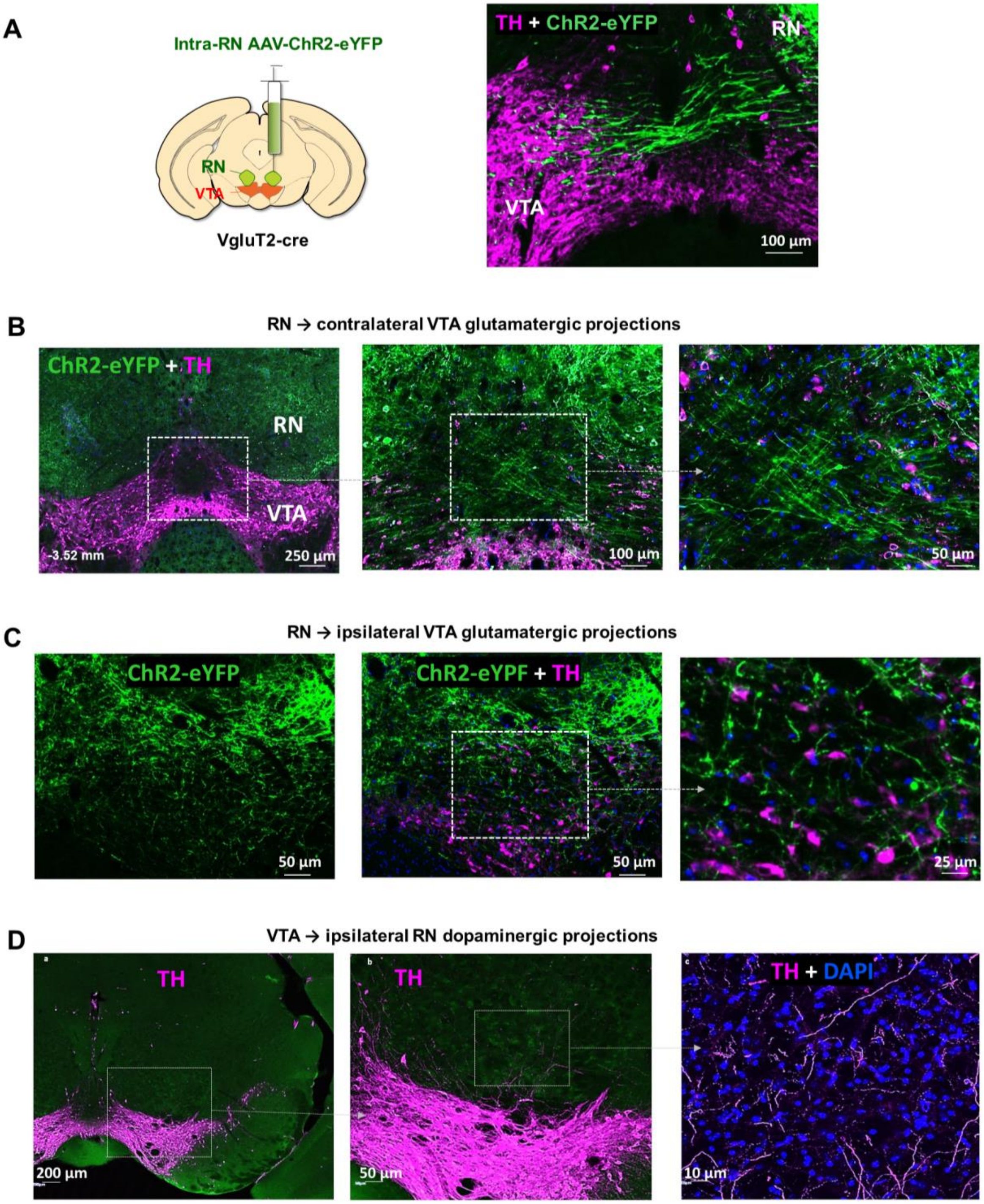

Figure S5 (related to Fig. 4): Bidirectional neural projections between the RN and VTA. ***A***: RNm-VTA glutamate projection as assessed by unilateral microinjection of AAV-ChR2-eYFP into the RNm. ***B***: AAV-ChR2-eYFP was injected into bilateral RNm to express ChR2-eYFP in glutamate neurons. The eYFP-labeled fibers (green) project from the RN to or go through the contralateral VTA; ***C***: The eYFP-labeled glutamatergic fibers also project from the RNm to the ipsilateral VTA; ***D***: TH-positive DA neurons project from the VTA to the ipsilateral RNm.

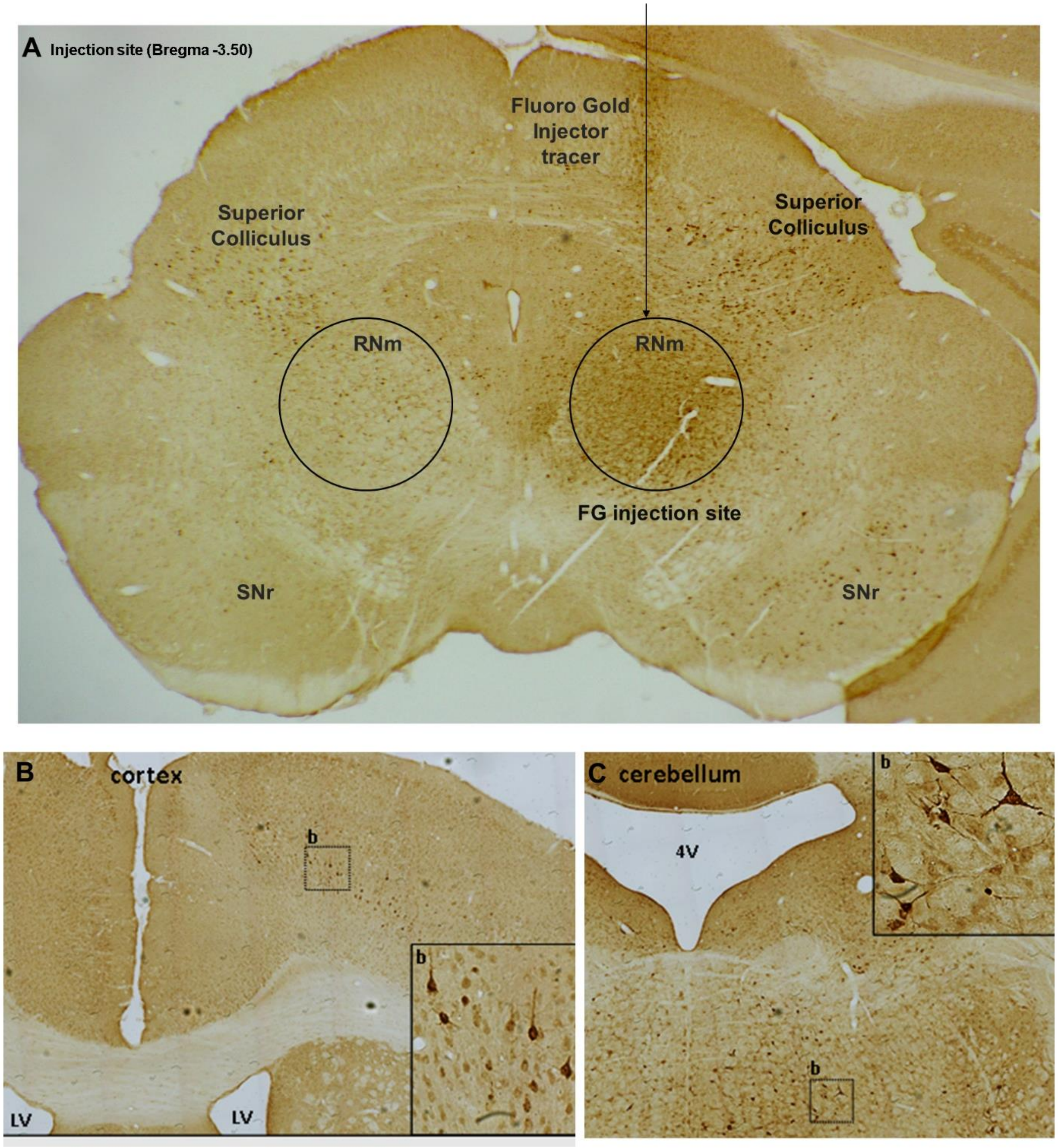

Figure S6 (related to Fig. 4 and Fig. 5): Fluoro-gold (FG) retrograde tracing of RNm afferents. ***A***: The classical retrograde tracer FG was microinjected into one side of the RNm. FG-labeled cells were detected in the ipsilateral SNr (substantia nigra pars reticulata), contralateral RNm, and bilateral superior colliculus; ***B/C***: FG-labeled cells were also found in the ipsilateral cortex (possibly motor cortex) and bilateral cerebellum.

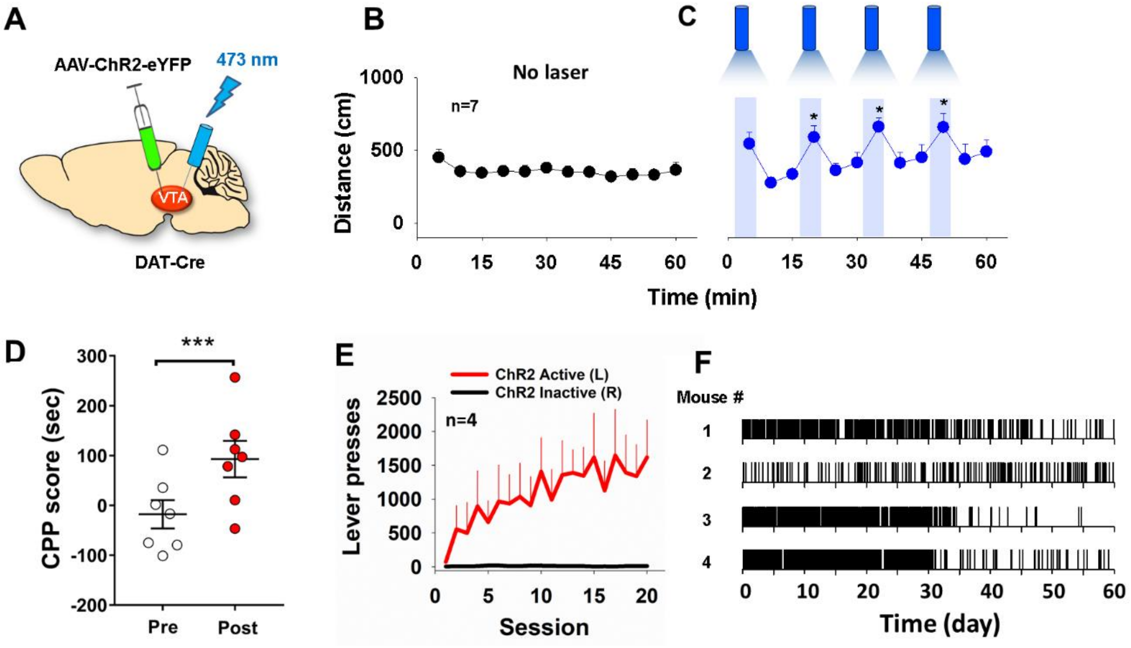

Figure S7 (related to Fig. 6): Optical stimulation of VTA DA neurons was both locomotor-stimulating and rewarding. ***A***: A schematic diagram showing that AAV-ChR2-eYFP was microinjected into bilateral VTA and the stimulation optrodes targeted bilateral VTA in DAT-cre mice; ***B/C***: Laser stimulation of VTA DA neurons increased open-field locomotor activity (one-way RM ANOVA, $F_{23,138} = 5.59$, $p<0.001$); ***D***: Laser stimulation of VTA DA neurons produced significant CPP in DAT-Cre mice (paired t-test, $p<0.001$); ***E***: Laser stimulation of VTA DA neurons produced robust oICSS behavior as assessed by active lever responses over inactive lever responses; ***F*****:** Representative oICSS responses (active lever presses) maintained by optical stimulation of VTA DA neurons (n=4). *$p<0.05$, ***$p<0.001$, as compared to baseline before laser stimulation (***C***) or pre-conditioning (***D***).

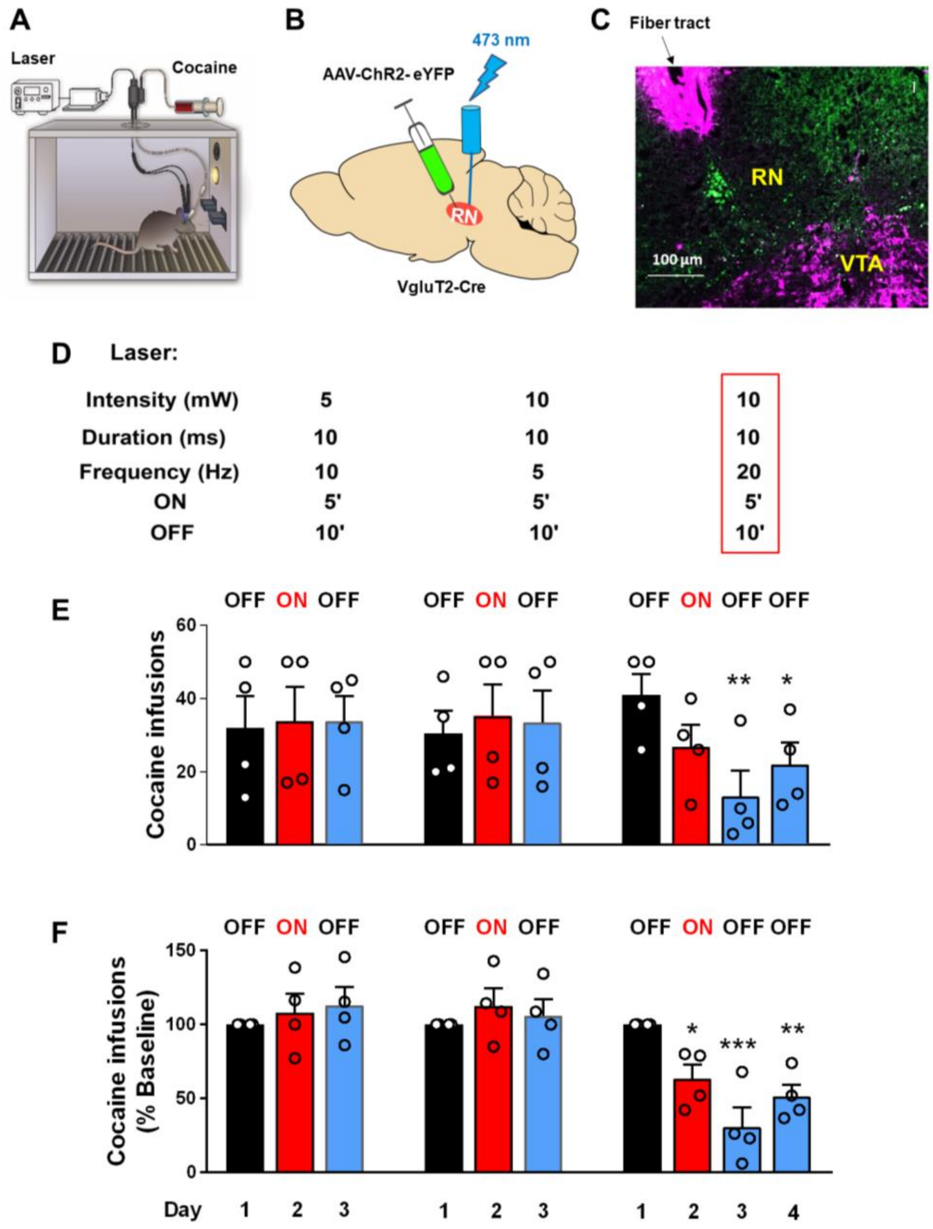

Figure S8 (related to Fig. 10): Determination of optimized stimulation parameters in cocaine self-administration. ***A***: A schematic diagram showing the experimental methods; ***B***: AAV-ChR2-eYFP was microinjected into the bilateral RNm and the stimulation optrodes targeted the bilateral RNm; ***C***: Representative images showing ChR2-eYFP (green) expression in the RNm; ***D***: Different stimulation parameters tested in cocaine self-administration; ***E/F:*** Optical stimulation of RNm glutamate neurons, with parameters (473 nm, 20 Hz, 10 ms pulse duration, 10 mW, 5-min laser ON followed by 10-min OFF), significantly inhibited cocaine self-administration without abnormal locomotor effects (***E***, right panel: one-way RM ANOVA, $F_{3,9} = 12.65$, $p=0.001$; ***F***, right panel: $F_{3,9} = 16.51$, $p<0.001$). $^*p<0.05$, $^{**}p<0.01$, $^{***}p<0.001$, compared to baseline on Day 1 (when laser was OFF).

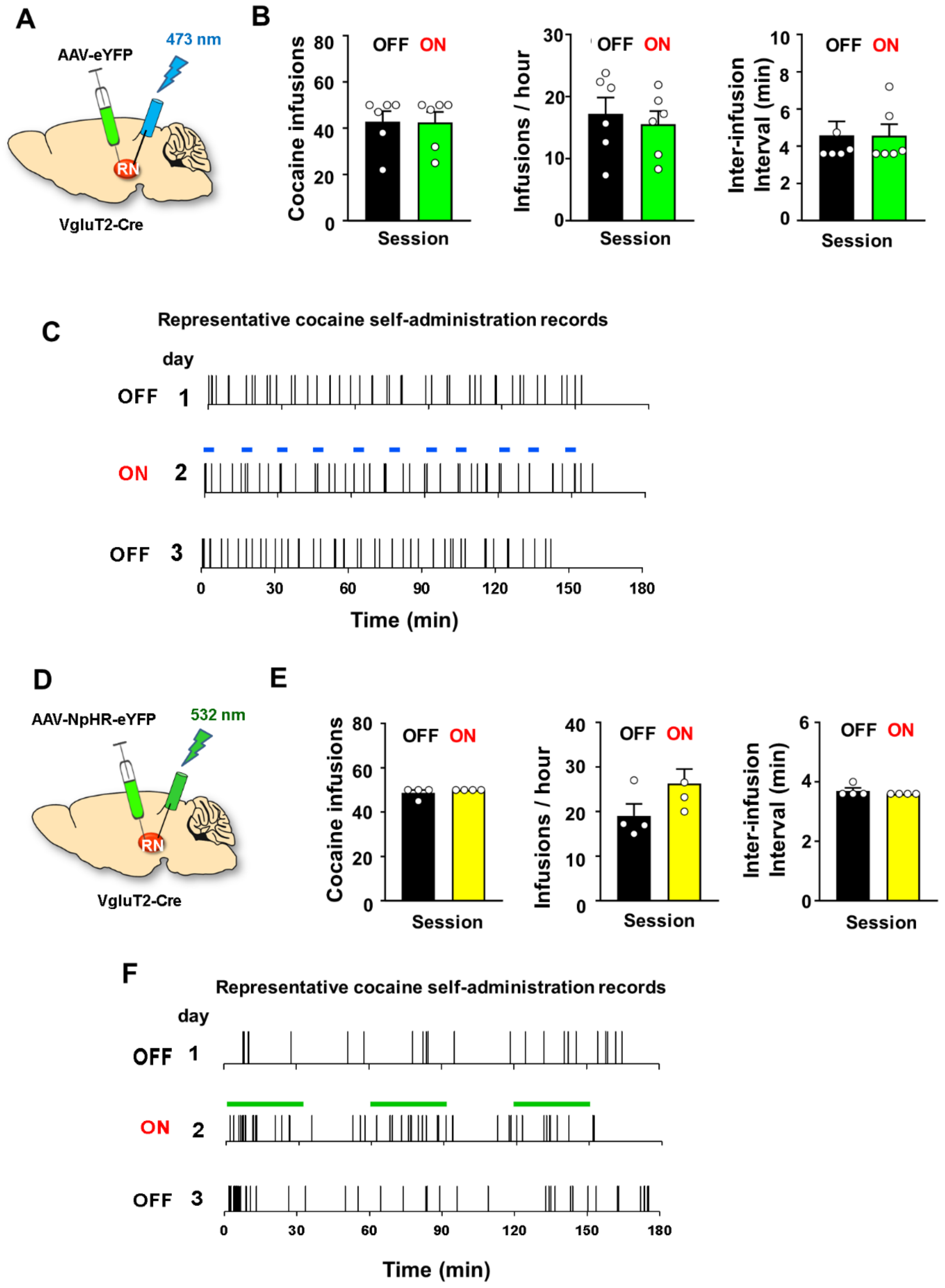

Figure S9 (related to Fig. 10): Effects of optical inhibition of RNm glutamate neurons on cocaine self-administration. ***A***: Schematic diagrams showing the general experimental methods. AAV- eYFP control virus was injected into the bilateral RNm, while the stimulation optrodes targeted the RNm; ***B***: Optical stimulation of the RNm failed to alter cocaine self-administration in mice having received the AAV-eYFP microinjections; ***C***: Representative cocaine self-administration records, illustrating that laser stimulation of the RNm had no effect on cocaine self-administration. ***D***: Schematic diagrams showing the general experimental methods. AAV-NpHR-

eYFP was injected into the bilateral RNm, while the stimulation optrodes targeted the RNm. ***E***: Optical inhibition of RNm glutamate neurons increased the rate of cocaine infusions but didn't alter the total number of cocaine infusions or averaged inter-infusion intervals. ***F***: Representative cocaine self-administration records, illustrating that optical inhibition of RNm glutamate neurons tended to increase cocaine self-administration.

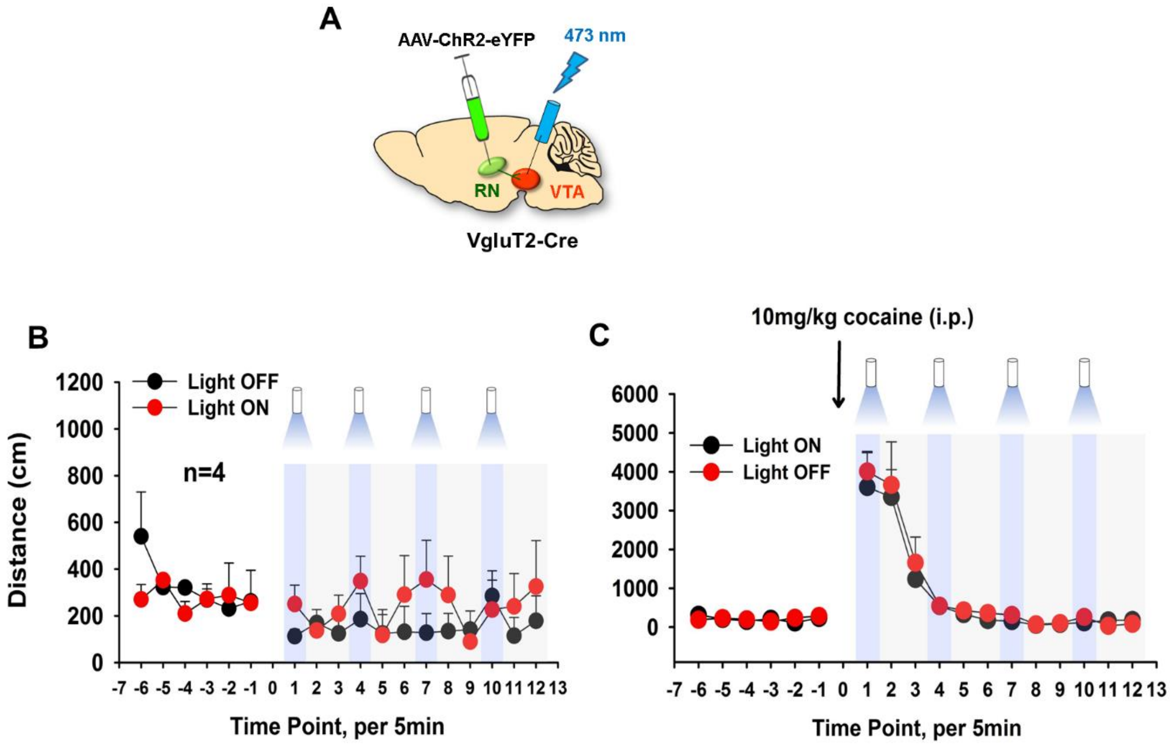

Figure S10 (related to Fig. 10): Effects of optical stimulation of RNm glutamate terminals in the VTA on basal or cocaine-enhanced locomotion. ***A:*** A schematic diagram showing the experimental methods; ***B***: Laser stimulation of RNm glutamate terminals failed to alter basal level of locomotion; ***C***: Laser stimulation of RNm glutamate terminals in the VTA also failed to alter cocaine-induced hyperactivity.

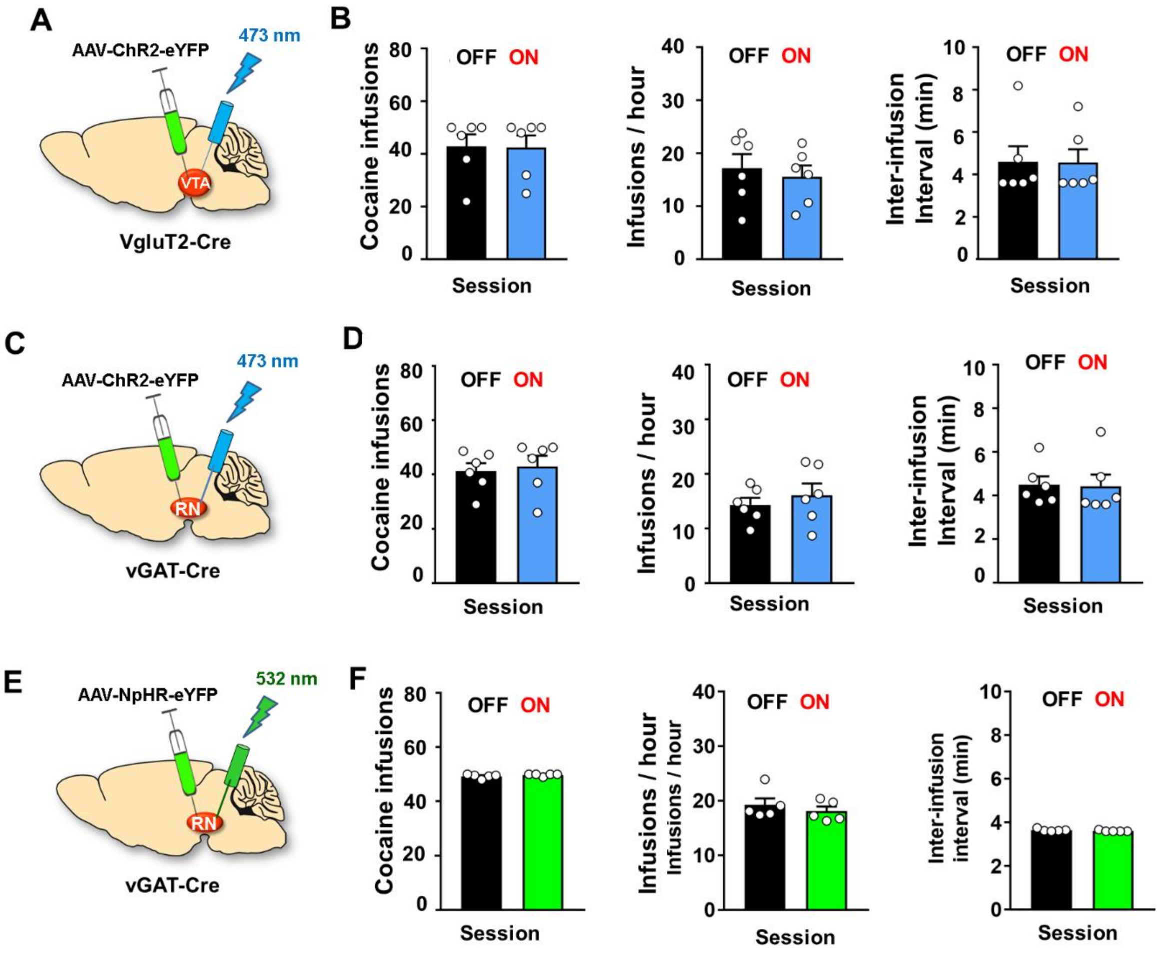

Figure S11 (related to Fig. 10): Effects of optical manipulations of VTA glutamate neurons or RN GABA neurons on cocaine self-administration. ***A/B***: Optical stimulation of glutamate neurons in the VTA failed to alter cocaine self-administration in VgluT2-Cre mice that had received AAV-ChR2-eYFP microinjections; ***C/D***: Stimulation of GABA neurons in the RNm failed to alter cocaine self-administration in vGAT-Cre mice that had received AAV-ChR2-eYFP microinjections; ***E/F***: Optical inhibition of GABA neurons in the RNm also failed to alter cocaine self-administration in vGAT-Cre mice that had received AAV-NpHR-eYFP microinjections.

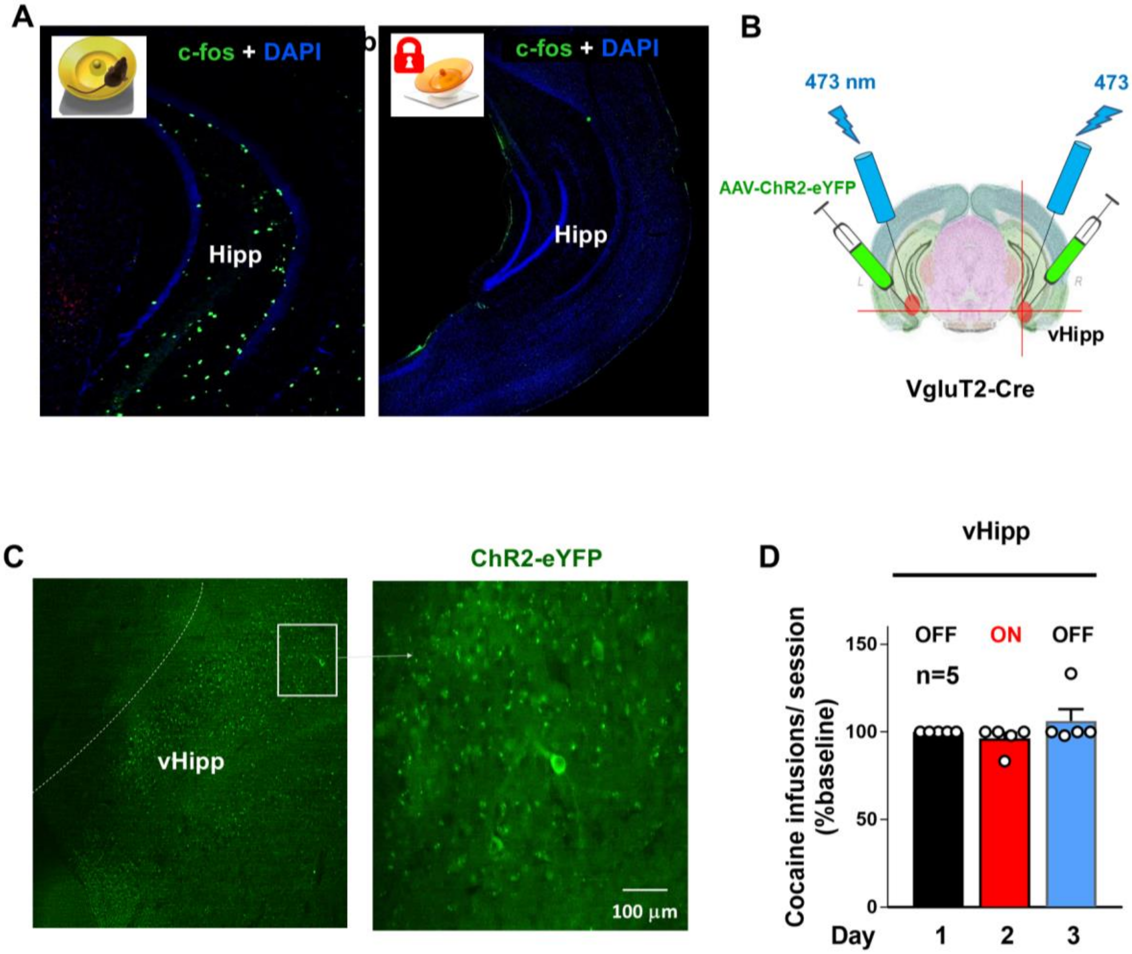

Figure S12 (related to Fig. 10). Effects of optical stimulation of glutamate neurons in the vHipp on cocaine self-administration. ***A***: Representative images showing c-fos expression in hippocampus in both wheel-running and wheel-locked mice. Wheel-running increased c-fos expression in the hippocampus. ***B***: A schematic diagram showing the experimental methods; ***C***: Representative images showing AAV-ChR2-eYFP expression in vHipp; ***D***: laser stimulation of glutamate neurons in vHipp failed to alter cocaine self-administration in VgluT2-Cre mice.
